# Reinforced Concrete Beams with FRP and Hybrid Steel–FRP Composite Bars: Load–Deflection Response, Failure Mechanisms, and Design Implications

**DOI:** 10.3390/ma18184381

**Published:** 2025-09-19

**Authors:** Paulina Dziomdziora, Piotr Smarzewski

**Affiliations:** 1 Faculty of Civil Engineering and Geodesy, Doctoral School of Military University of Technology, 2 Gen. Sylwestra Kaliskiego, 00-908 Warsaw, Poland; 2 Faculty of Civil Engineering and Geodesy, Military University of Technology, 2 Gen. Sylwestra Kaliskiego, 00-908 Warsaw, Poland

**Keywords:** reinforced concrete beams, fiber-reinforced polymer (FRP) bars, steel–FRP composite bars (SFCBs), hybrid reinforcement, load–deflection response, ductility and serviceability, failure mechanisms, durability and aggressive environments, design codes

## Abstract

Corrosion concerns motivate the use of alternatives to conventional steel reinforcement in RC beams. This review evaluates fiber-reinforced polymer (FRP) bars and hybrid steel–FRP composite bars (SFCBs) used for durability-critical applications. We conducted a structured literature search focused on 2010–2025 and included seminal pre-2010 studies for context. Experimental studies and code provisions were screened to synthesize evidence on load–deflection response, cracking, and failure, with brief notes on UHPC systems. FRP-RC offers corrosion resistance but limited ductility and an abrupt post-peak response. Steel is ductile and provides warning before failure. SFCB combines durability with steel-core ductility and yields gradual softening and higher energy absorption. Practice should select reinforcement based on stiffness–ductility–durability trade-offs. Current codes only partially cover hybrids. Key gaps include standardized bond–slip and tension-stiffening models for SFCB and robust data on long-term performance under aggressive exposure.

## 1. Introduction

The durability of reinforced concrete structures is a key aspect of civil engineering, especially in the context of increasing requirements for infrastructure operation under aggressive environmental conditions, such as high humidity, the presence of chlorides, and the use of de-icing salts. One of the main factors limiting the service life of these structures is the corrosion of the steel reinforcement, which leads to the deterioration of the mechanical properties of the bars and weakening of the bond between steel and concrete [1]. This results in costly renovation work and a shorter service life of the structure [2,3,4].

In response to this problem, there is growing interest in polymer composite bars, which, thanks to their high strength, light weight, and corrosion resistance, constitute a viable alternative to traditional steel bars in highly aggressive environments, such as coastal regions or industrial facilities [5,6,7,8,9,10,11,12,13,14,15,16,17].

Fiber-reinforced polymer (FRP) composites consist of reinforcing fibers and a resin matrix, which gives them unique mechanical and operational properties [18,19,20]. The most commonly used include carbon fiber-reinforced polymer (CFRP), glass fiber-reinforced polymer (GFRP), aramid fiber-reinforced polymer (AFRP), and basalt fiber-reinforced polymer (BFRP). The introduction of continuous fibers, as well as the hybridization of different fiber types, has attracted significant interest due to the improved mechanical properties such as higher stiffness, strength, and better resistance to dynamic loads. Such hybrid solutions have shown promising results in overcoming the limitations of short fibers, allowing for better performance in structural applications [21]. BFRP offers a favorable compromise between strength, a higher modulus of elasticity compared to GFRP, better thermal stability, and lower environmental and economic costs compared to CFRP and AFRP [22,23,24,25,26,27,28,29,30]. The use of FRP as internal reinforcement eliminates the problem of corrosion, making it an attractive solution in modern concrete structures. FRP bars, laminates, and profiles are used in civil engineering, hydraulic engineering, and transportation construction, among others [31,32,33,34,35,36,37,38,39,40,41,42,43,44,45,46,47]. Their main advantages include high tensile strength and chemical resistance [48,49]. Furthermore, their use is consistent with efforts to reduce the environmental impact of construction, where both cement and steel production are associated with high greenhouse gas emissions. Due to their durability and low carbon footprint, FRP can contribute to more sustainable development [50,51]. Corrosion resistance is the most important advantage of FRP. It effectively prevents reinforcement degradation under aggressive environmental conditions [16,18,52,53,54,55]. In addition, the development of 3D printing technologies, such as FDM (Fused Deposition Modeling), allows for the manufacturing of complex FRP composite structures at a lower cost, without the need for specialized tools. This technology has demonstrated potential for producing high-performance parts, particularly in industries requiring lightweight components such as aerospace and automotive engineering, as well as in structural applications [21]. FRP is characterized by a very high tensile strength of 483 MPa to 3689 MPa, low weight, lack of magnetic properties, and good fatigue resistance [18,47,56,57]. Environmental studies indicate that the durability of FRP depends on the type of fiber and matrix; for example, polyester resin is less resistant to exposure than epoxy resin. Standards introduce reduction factors for the design strength of FRP depending on the environment (so-called environmental reduction factors).

However, FRP composites also have limitations. They exhibit linear elastic characteristics until failure and are incapable of plastic deformation, resulting in sudden and brittle failure [58,59,60]. Their modulus of elasticity, in the range of 35–125 GPa for AFRP, BFRP, and GFRP and 120–580 GPa for CFRP, corresponds to only 25–75% of the modulus of steel [18,47,57,61]. As a result, FRP-reinforced concrete elements are characterized by greater deflections, wider cracks in the service state, and lower stiffness after concrete cracking [13,23,47,62,63,64,65,66,67,68,69]. Another challenge is the limited ductility resulting from the inability of the material to yield, which limits the structure’s ability to dissipate energy and its resistance to dynamic loads [70,71,72,73,74]. FRPs also exhibit lower shear and compressive strength and sometimes insufficient adhesion to concrete due to their anisotropic structure and the possibility of micro-buckling of fibers [75,76,77,78,79]. Despite research developments, the fire resistance of FRP remains an issue to be resolved [80,81,82,83,84,85,86,87].

To overcome these limitations, steel fiber composite bars (SFCBs) were developed that combine the advantages of both materials. They consist of an inner steel core and an outer FRP shell, providing reinforcement with high strength, good ductility, corrosion resistance, and improved durability [1,51,88,89,90]. The FRP shell protects the steel core from corrosion, and its fibers, usually longitudinally oriented, participate in load transfer. Under the action of tensile forces, SFCBs exhibit two-stage behavior: initial linear elasticity until steel yields, followed by a hardening phase, which is important for structural safety and prediction of damage [91,92]. Due to this design, SFCBs offer a higher initial modulus of elasticity than conventional FRP, better ductility than steel, and greater environmental resistance. In addition, they exhibit higher secondary stiffness after yielding and greater energy dissipation capacity under cyclical loading [90,91,93,94,95,96]. SFCBs are manufactured industrially, primarily by pultrusion. This process involves preparing a steel core, impregnating FRP fibers with resin, and then forming and curing them in a special matrix [97]. Depending on the fibers used, various types of SFCBs are distinguished: steel–basalt fiber composite bar (SBFCB), steel–glass fiber composite bar (SGFCB), steel–carbon fiber composite bar (SCFCB), and steel–aramid fiber composite bar (SAFCB) [51,98,99,100]. At the same time, hybrid reinforcement concepts are being developed, which involve the simultaneous use of steel and FRP bars in various configurations. By properly selecting the degree of reinforcement and material arrangement, it is possible to achieve an optimal compromise between the durability and ductility of the structure [49,56,89,101,102].

This review provides a comprehensive, up-to-date synthesis of how composite reinforcement affects the mechanical behavior of RC members. It compares steel, FRP, and SFCB in a unified load–deflection framework with emphasis on post-peak response and serviceability, synthesizes head-to-head trends at matched geometry and reinforcement ratio using normalized changes, treats UHPC/ECC as contextual with stated transfer limits, and integrates durability evidence and code provisions while pinpointing hybrid-specific gaps in bond–slip and tension-stiffening.

## 2. Methodology

This review article adopted a systematic approach to ensure the reliability and transparency of the research process. In the first stage, an extensive literature search was conducted in reputable scientific databases, such as ScienceDirect and Scopus using carefully selected keywords. The search focused on publications from 2010 to 2025, with seminal pre-2010 works also included for context. The initial search yielded a large number of records. After screening, approximately 80+ publications were shortlisted as directly relevant, including both experimental and review studies that informed the sections on RC beams with different reinforcement types and on the mechanical and durability properties of the bars.

The publication selection process was conducted in several stages. First, titles, abstracts, and keywords were analyzed, eliminating articles that were not directly related to the topic or exhibited a low level of scientific credibility. Only publications containing experimental results on the mechanical properties of beams reinforced with composite bars and/or fibers and composite bars were included in the further analysis. The eligible articles were then subjected to a detailed content analysis.

**Figure 1:**
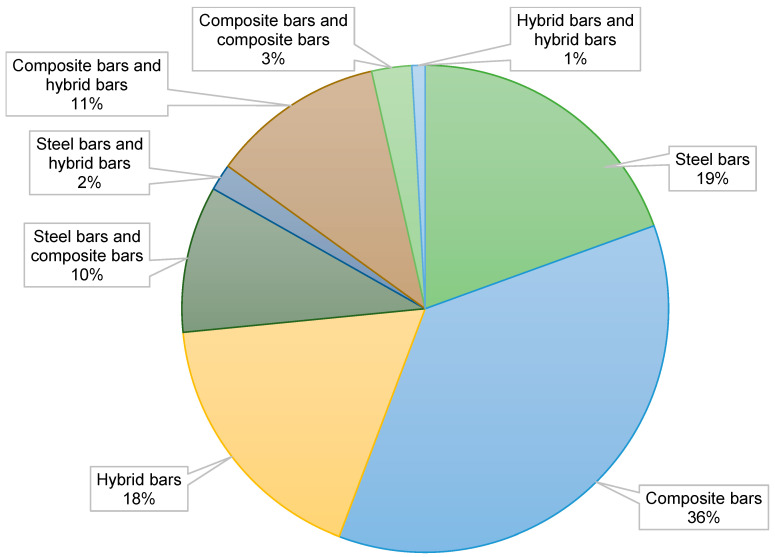
Research topic in the available literature according to the type of reinforcement used.

**Figure 2:**
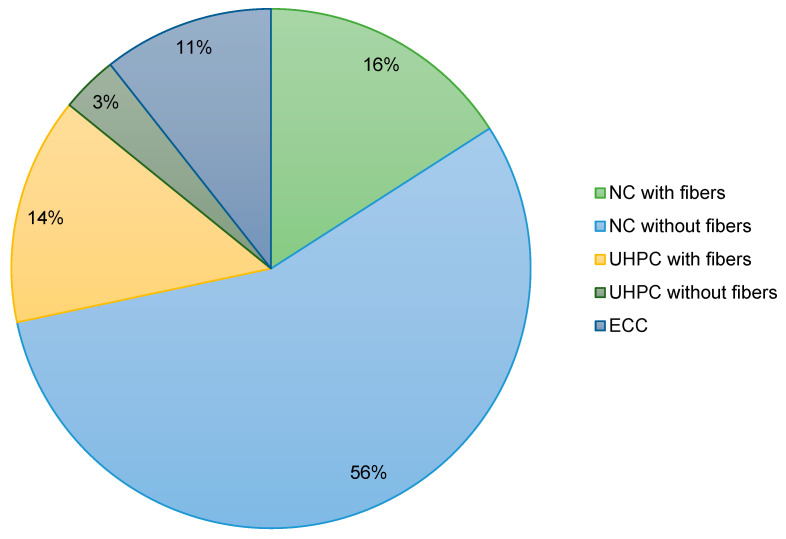
Research topics in the available literature according to the type of concrete used (Note: NC—normal concrete).

The articles were classified according to the type of reinforcement used (Figure 1), the type of concrete used (Figure 2), and the scope of the research. Particular attention was paid to the analysis of strength parameters, durability, and failure mechanisms. In reference to Figure 1, which shows the classification of publications according to the type of reinforcement, it can be seen that the majority of studies focus on composite bars (36%), while steel bars make up 19% of the publications. Hybrid bars are less commonly studied, constituting 18% of the research. Meanwhile, Figure 2, which presents the classification of publications by the type of concrete used, shows that normal concrete both with fibers 16% and without fibers 56%, dominates the research. Smaller groups are formed by studies on UHPC and ECC, indicating that these areas are still niche in the literature. Based on the materials collected, a comparative analysis of the mechanical properties of various types of FRP bars was conducted, the impact of composite reinforcement on the load-bearing capacity of concrete elements was assessed, and structural failure mechanisms were compared depending on the type of reinforcement used. In addition, a statistical analysis was conducted that allowed us to determine the frequency of specific research topics in the available literature and identify areas that require further research.

## 3. Characteristics of Reinforcement of Structural Elements

### 3.1. Classification of Reinforcing Materials

**Figure 3:**
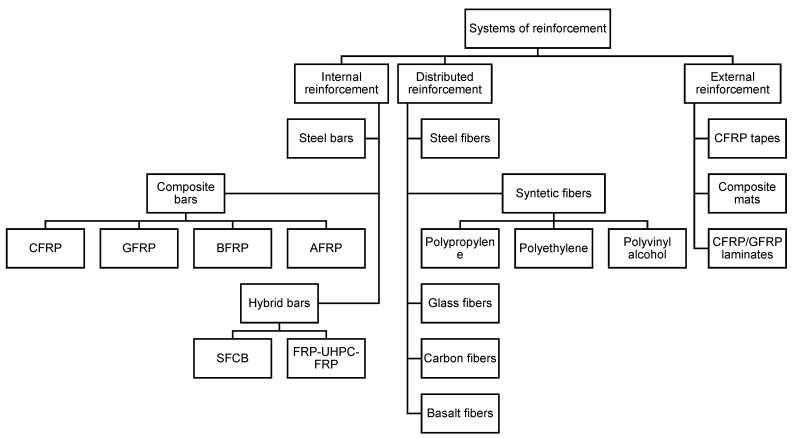
Classification scheme of reinforcement types.

Reinforcement is an essential element of modern concrete structures, as it allows effective transfer of tensile stresses and improves the mechanical properties of concrete. Depending on the type of structure, its intended use, operating conditions, and construction technology, various types of reinforcement are used. One of the basic criteria for their classification is the position of the reinforcement material relative to the concrete matrix. In this context, three main types of reinforcement are distinguished (Figure 3). The first is internal reinforcement, which involves embedding bars or meshes in the concrete. The second type is dispersed reinforcement, which involves adding reinforcing fibers directly to the concrete mix. The third category is external reinforcement, which involves reinforcing prefabricated structural elements with composite materials, such as FRP tapes or mats, bonded to the concrete surface. Each of these types of reinforcement serves a different function and is used in various construction and design situations.

### 3.2. Mechanical and Physical Properties

#### 3.2.1. Steel Bars

Steel bars, whose strength parameters are summarized in Table 1, are characterized by a Young’s modulus of approximately 200 GPa, which translates into high material stiffness. Their yield strength ranges from 240 to 600 MPa and maximum stresses range from 350 to 705 MPa [49,50,51,89,101,102,103,104,105,106]. The advantages of this type of bar include predictable load behavior, familiar manufacturing technology, and the ability to plastically deform before failure. Their main disadvantages are their susceptibility to corrosion, high weight, and limited chemical resistance.

Traditional steel bars remain the cheapest option in terms of unit cost. A 10 mm diameter steel bar typically costs between $0.80 and $1.10 per meter. However, this price does not include the cost of corrosion protection (e.g., epoxy coating) or potential repair and maintenance costs resulting from corrosion during use. However, steel has the advantage of a well-developed recycling infrastructure and easy availability.

#### 3.2.2. Composite Bars

Based on the analysis of the data in Table 2, two main groups of composite rods can be distinguished based on the reinforcing material used: carbon fiber rods and glass fiber rods. Composite rods often lack a classic yield point but fracture after reaching maximum deformation. In addition to fiber type, the mechanical properties of these rods are influenced by factors such as fiber-to-resin ratio, matrix composition, and surface morphology. For instance, CFRP rods typically have a higher fiber-to-resin ratio, contributing to their exceptional stiffness and tensile strength. Surface treatments such as ribbing or sand-coating further enhance bond strength with concrete, improving load transfer efficiency [49,50,104]. Carbon fiber composite bars [102,105] are characterized by very high Young’s modulus values compared to glass rods, ranging from 125 to 192 GPa. This demonstrates the exceptional stiffness of these materials, which in some cases reaches 96% of the stiffness of a steel rod. Additionally, they exhibit very high tensile strength, ranging from 2001 MPa to 2701 MPa. Their high mechanical properties make carbon fiber rods ideal for applications requiring high load-bearing capacity and stiffness, such as bridge structures or reinforcement elements in extreme conditions. In terms of material composition, CFRP bars are typically made using continuous carbon fibers embedded in a polymer matrix, often epoxy or vinyl ester. Studies [102] indicate that even within the same fiber volume fraction, variations in rib depth (e.g., 0.2 mm vs. 0.6 mm) can lead to significant differences in mechanical interlock and bond strength, due to changes in surface morphology. The ribbed or sand-coated textures enhance the mechanical interlock between the bar and concrete matrix, reducing slip and improving stress transfer. Their common disadvantage is the high production cost resulting from the price of carbon fiber. CFRP rods are significantly more expensive than GFRP rods and steel rods. Carbon fiber as a raw material costs between $20 and $33 per kilogram. In practice, this means that 10 mm diameter CFRP can cost from $5 to even $15 per meter, with larger diameters reaching prices exceeding $20/m. These high costs result from the energy-intensive and complex production and processing of carbon fiber. For this reason, CFRP is mainly used in specialized projects requiring very high strength at low weight, such as in bridge infrastructure or structures exposed to very aggressive environments. CFRP contributes the highest carbon emissions per unit of mass and volume. The results of most studies indicate a growing consensus on this issue [107,108,109]. Furthermore, different matrix types can result in different carbon emission factors of FRP. Resin has been shown to be the largest contributor to carbon emissions from FRP tendons, with polyester resin having a slightly higher emission factor than epoxy [110]. Therefore, the FRP matrix should be carefully selected during the design phase of sustainable structures. The carbon emission factor of CFRP is the highest compared to BFRP, GFRP, and steel bars. BFRP and GFRP have similar carbon emission factors to steel bars on a per unit mass basis, but volume-based comparisons show that BFRP has the lowest carbon emission factor, making it a more sustainable material from an environmental perspective [107].

In contrast, glass fiber composite bars [49,50,101,104,106,111] exhibit lower Young’s modulus values ranging from 40 GPa to 64 GPa, which corresponds to 20–32% of the stiffness of a steel bar. Despite this, their tensile strength remains high, ranging from 520–1000 MPa, making them competitive with many conventional reinforcing materials. The GFRP bars used in studies were composed of E-glass fibers embedded in vinyl ester or polyester resin matrices, with a typical volumetric fiber content of about 64% [49]. Various manufacturing methods have been employed, including manual filament winding [101] and pultrusion [49], with surface modifications such as ribbing or sand-coating [105,111] to enhance bond strength. For example, GFRP bars with ribbed surfaces formed during pultrusion demonstrated improved interfacial behavior with concrete, reducing bond-slip and enhancing structural integrity. Glass fibers have the advantage of being more flexible than carbon fibers and are less expensive, which supports their widespread use in construction. GFRP bars are typically 2–3 times more expensive than traditional steel rebars, but their unit price can be similar or even lower for smaller diameters. The average cost of a 10 mm diameter bar ranges from approximately $0.45 to $1.80 per meter, depending on the manufacturer, diameter, and country. The production cost of the material itself is approximately $0.15/m, including resin, fiberglass, and energy consumption [111]. GFRP is particularly cost-effective in applications requiring corrosion resistance, as it requires no anti-corrosion treatment, which reduces operating costs. In terms of carbon dioxide emissions, GFRP achieves emission factors comparable to steel bars, making it a more environmentally friendly choice than CFRP.

#### 3.2.3. Hybrid Bars

Based on the test data presented in Table 3, obtained by Sun et al. [90] and Huang et al. [51], it can be seen that hybrid bars with a steel core and an outer basalt fiber are characterized by good tensile strength, with a maximum stress reaching 939 MPa. Young’s modulus ranges from approximately 105 to 175 GPa, which corresponds to 50–87% of the modulus of the steel bar. The advantages of basalt fibers include their corrosion resistance and moderate cost, making them an attractive compromise between carbon fiber and glass fiber. The disadvantage is their lower stiffness compared to carbon fiber. Increasing the number of fiber layers around the core improves strength but simultaneously reduces the modulus of elasticity [101]. Studies by Etman et al. [89] and Huang et al. [51] demonstrated that rods with a steel core and an outer carbon fiber core achieved the best mechanical properties among the analyzed solutions. Young’s modulus reaches almost 200 GPa, making these rods very stiff and comparable, and often even superior, to steel rods. Tensile strength can reach up to 1620 MPa [51], which is higher than for other materials. The disadvantages of such rods are their brittleness and high cost. Due to their high stiffness and strength, they are particularly useful in structures requiring minimal deflection and high loads. Studies by Abdel-Karim et al. [101], Huang et al. [51], Liu et al. [112], and Zhang et al. [113] indicate that rods made with a steel core and an outer layer of glass fiber are the cheapest and most readily available option. Their tensile strength ranges from approximately 700 to 766 MPa, and the modulus of elasticity reaches a maximum of approximately 140 GPa, although it is more often significantly lower. Their low stiffness causes these rods to deflect more, making them better suited for auxiliary structures or where deformation is not critical. Due to their low stiffness, these rods are characterized by greater deflection and should therefore be used in auxiliary structures or where greater deformation is permissible. Their significant advantages include high chemical resistance and lack of susceptibility to corrosion.

Studies by Liu et al. [112] and Zhang et al. [113] presented rod designs whose cores are made of glass fibers, with an annular UHPC layer and an outer sheath of glass fibers (GFRP-UHPC-GFRP) or carbon fibers (GFRP-UHPC-CFRP), and carbon fibers, with an annular UHPC layer and an outer sheath of glass fibers (CFRP-UHPC-GFRP). Such rods eliminate the corrosion problem, are very lightweight, and their tensile strength can be very high, up to 2296 MPa in the case of a carbon core [112]. However, their Young’s modulus remains low, around 44 GPa, which means limited stiffness and greater deflections under load.

The costs of hybrid bars are more difficult to determine definitively as they depend on the manufacturing technology and the proportions of the materials used. However, it is generally accepted that the cost of such a rod may be slightly higher than that of a standard steel rod, but significantly lower than that of GFRP or CFRP. The hybrid structure maintains good load-bearing capacity due to the steel core and offers increased corrosion resistance due to the glass, basalt, or carbon fiber coating. This solution can offer a cost-effective compromise between durability, price, and recyclability. Using a thin composite coating, it is possible to significantly reduce the use of high-carbon composite material while simultaneously increasing corrosion resistance compared to a steel rod. The environmental impact of such rods depends primarily on the material composition. The steel core can be easily recycled, with a recovery rate of up to 85%, which positively impacts the environmental balance at the end of the structure’s life cycle. A thin layer of glass or basalt fiber, on the other hand, provides protection without significantly increasing the rod’s weight or causing a significant increase in CO_2_ emissions. A carbon fiber coating would be less environmentally beneficial due to the aforementioned production emissions. These types of rods could be particularly interesting from a sustainable construction perspective, as they combine the strength and durability of composites with the recyclability and economics of steel.

#### 3.2.4. Discussion

**Figure 4:**
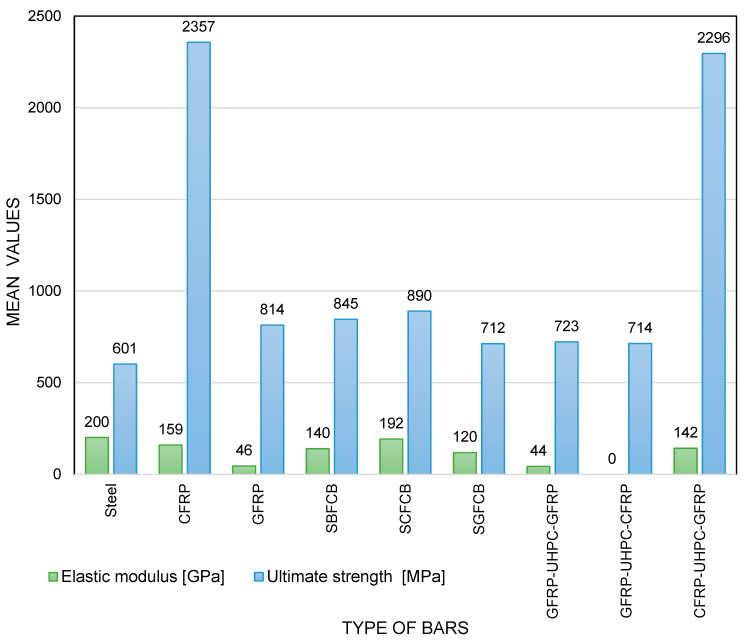
Comparison of mechanical properties of reinforcement bars. Bars show literature means. Min–max ranges are given in Table 4. Values depend on grade/fiber–resin system, diameter, surface profile, and test method.

**Figure 5:**
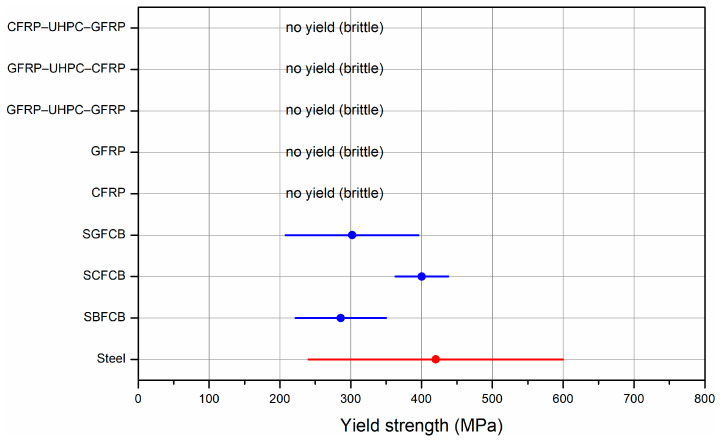
Ranges of yield strength (min–max) for steel and SFCB (mid-points shown). FRP and UHPC-based hybrids: no yield (brittle). Data summarized in Table 4.

Based on the analysis of the mechanical parameters summarized in Table 4 and the economics of the various types of structural bars, it can be seen that they share common characteristics and significant differences that determine their suitability for specific applications. All of the bars discussed were designed to reinforce the structures and carry heavy loads. A common feature is high tensile strength and the need to balance cost, durability, and environmental impact. Despite their common structural function, the differences between these materials are significant and primarily relate to stiffness, strength, price, and carbon footprint as well as material composition and surface morphology. The mechanical properties of composite and hybrid bars are strongly influenced by factors such as the fiber-to-resin ratio and the texture of their surface, which affect bond strength with concrete and load transfer efficiency. A comparison of the various types of reinforcement is presented in Figure 4 and Figure 5.

The steel bar has the highest modulus of elasticity of all the bars analyzed and an average tensile strength of 600 MPa. Compared to the steel bar, CFRP has an elastic modulus of approximately 20% lower, but its tensile strength is almost four times higher, reaching 2700 MPa. This can be attributed to the high fiber-to-resin ratio typical for CFRP, which significantly improves both stiffness and tensile strength. The surface of CFRP bars, often treated with ribbing or sand-coating, enhances the bond with concrete, improving the overall structural integrity and load transfer [49,50,104]. GFRP has an elastic modulus 75% lower than the steel bar, but its strength is 38% higher. SBFCB has an elastic modulus 30% lower than the steel bar and 41% higher strength. SGFCB, on the other hand, has an elastic modulus slightly lower than the steel bar and 48% higher strength. Among the composite bars, CFRP clearly stands out, possessing the highest strength of all the bars analyzed and the second-highest modulus of elasticity, 17% lower than SGFCB. SGFCB achieves the highest modulus of elasticity among the bars, with a strength of 890 MPa, 289% lower than CFRP. Among the GFRP-UHPC-GFRP hybrid bars, it has the lowest modulus of elasticity and strength of all the bars. CFRP-UHPC-GFRP, on the other hand, clearly outperforms the other hybrids, achieving an average strength of 2296 MPa, which is 222% higher than the steel bar and 3% lower than CFRP, and a modulus of elasticity 29% lower than the steel bar. This hybrid bar combines the high strength of CFRP with the corrosion resistance and cost-effectiveness of GFRP, making it a versatile option for structures requiring both strength and durability at a reduced cost compared to full CFRP bars [51,101,112,113]. Comparing fiber-core hybrid rods to steel-core hybrid rods, CFRP-UHPC-GFRP, despite having an elastic modulus 11% lower than SBFCB and 26% lower than SGFCB, retains a high level of strength similar to CFRP. In contrast, GFRP-UHPC-GFRP and GFRP-UHPC-CFRP have elastic moduli significantly lower than all composite rods and strength comparable to GFRP.

From a practical construction application perspective, steel bars, due to their superior stiffness and easy availability, are best suited for structures requiring limited deformation, such as beams and columns in heavily loaded structures. CFRP, because of its exceptionally high strength and corrosion resistance, can be particularly useful in bridge structures, components exposed to marine environments, and in special structures where the structure’s weight must be kept as low as possible. Despite its low stiffness, GFRP can be used in chemically aggressive environments and in areas where greater deflections are permissible, such as foundations and water infrastructure components. SBFCB and SGFCB offer a compromise between corrosion resistance and stiffness, making them an alternative to steel bars in pedestrian bridges, piers, and bridge barriers, particularly in areas exposed to deicing salts. Among hybrid bars, CFRP-UHPC-GFRP combines high strength with moderate stiffness, making it a cost-effective choice for components that require high load-bearing capacity and durability, while offering a cost reduction compared to CFRP. GFRP-based hybrids, such as GFRP-UHPC-GFRP and GFRP-UHPC-CFRP, can be used in less demanding structures with increased durability where high stiffness is not necessary. The surface textures of these hybrid bars, often ribbed or sand-coated, enhance their bond strength with concrete, improving overall structural performance [51,101,112,113]. The main conclusion of the analysis is that the selection of the appropriate type of structural bar should be based on the specific project, operating environment, and technical and economic requirements. There is no universal solution that surpasses steel bars in all aspects, but there are materials available that offer better properties and durability under certain conditions.

### 3.3. Durability and Long-Term Use

The degradation mechanisms of composite bars differ significantly from those observed in steel bars. FRPs consist of fibers and a polymer matrix, and their durability is determined by many factors, such as the type and properties of the fiber, the type of resin matrix used, the quality of the bond at the interface and environmental conditions. This section discusses the research results, summarized in Table 5, on their resistance to temperature, alkaline and chloride environments, freeze–thaw cycles, and creep cracking. The analysis is based on numerous sources in the literature and experimental results, allowing for a comprehensive look at the degradation mechanisms of FRP under various operating conditions [107].

Exposure to high temperatures significantly affects the mechanical properties of FRP composite bars. The degradation observed in GFRP under high temperatures can be classified into clear stages:

- GFRPs exhibited distinct color and structural changes upon exposure to heat. At 200 °C, the GFRPs, initially green, turned brown and at 300 °C, intense carbonization occurred. At 400 °C, the resin was almost completely carbonized, exposing the glass fibers in the GFRP [43]. These phenomena correlated with significant mass loss, reaching 15–20% of the original mass, indicating resin degradation, especially after exceeding 300 °C.
- The failure modes of the rods were observed, from full failure in both shear planes to partial delamination, regardless of the rod diameter [78].
- Shear strength generally decreased with increasing rod diameter and was higher for BFRP than for GFRP at room temperature. Interestingly, after exposure to temperatures up to 300 °C, this strength initially increased, which is attributed to additional resin curing, and then rapidly decreased due to its decomposition [82].
- At 350 °C, the GFRP retained 50–80% of their shear strength, but after 400 °C–only 20–30%. In contrast, the compressive strength degraded more rapidly: 25 mm diameter GFRPs lost over 80% of their strength at 350 °C, and virtually all of it after 400 °C. This is due to the dominant role of the resin in compressive load-bearing capacity, and its decomposition leads to a dramatic loss of properties [43].

FRPs exposed to alkaline environments showed varying levels of durability:

- CFRPs retained 75% of their strength even after prolonged soaking in aggressive conditions, thanks to the impermeability of carbon fibers [114].
- BFRPs also exhibited good resistance, with strength retention values of 85–95%, outperforming GFRPs, which were most susceptible to chemical degradation, primarily due to the reactivity of glass with alkaline solutions [115].
- The protective resin layer was crucial, as its damage led to accelerated corrosion of the GFRP fibers.
- CFRPs were also resistant to acids [114]. Although BFRPs were inferior to CFRPs, they were often recommended as an alternative due to their favorable cost-effectiveness [116].
- CFRP and BFRP also outperformed GFRP in terms of resistance. GFRP showed strength retention of only 30–70% of the initial value, while CFRP and BFRP retained more than 70%. The performance of CFRP was up to 160% higher than BFRP under the most aggressive conditions [35,109].

Creep rupture is another critical issue for FRP composites under long-term loading:

- CFRP exhibits superior creep resistance.
- BFRP can outperform GFRP in long-term strength at lower stress levels [107].

The effect of freeze–thaw cycles on FRP bars varies by material type:

- The elastic modulus remained stable or even increased in most cases, while the tensile strength increased only for BFRP.
- GFRP and CFRP showed a decrease in tensile strength. The elongation at break generally degraded.
- BFRP exhibited the greatest instability resulting from changes in the elastic modulus [117,118].

**Figure 6:**
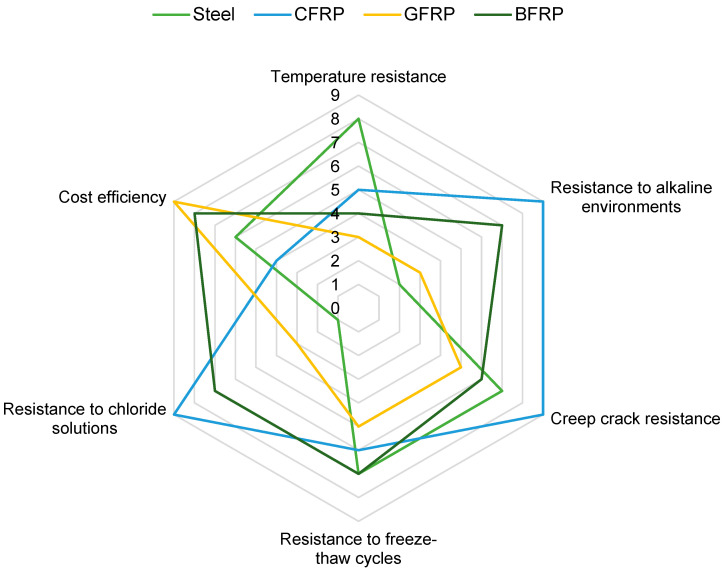
Comparison of the durability characteristics of composite bars.

In summary, the durability of FRP depends on many factors, as shown in Figure 6. CFRP demonstrated the highest resistance in almost all aspects tested, while BFRP presented a good balance between mechanical properties and environmental resistance. GFRP, although the cheapest, was the most susceptible to degradation, especially in alkaline and chloride-containing environments, which limits its use in aggressive operating conditions. In light of the research to date, future research should focus on further improving the properties of resins, which are a key element that influences FRP degradation. It seems important to develop methods for modifying the surface of the rods to improve the adhesion between the fibers and the matrix, which could reduce the delamination of the material under extreme conditions. It is also worth investigating the effect of hybrid reinforcement systems, combining different types of fiber to obtain a material with an optimal balance between mechanical strength and resistance to environmental factors. Further research on the mechanisms of creep fracture under simultaneous thermal and chemical loading, as well as the development of predictive models, will allow a better prediction of the long-term performance of structures [107,119].

## 4. Beam Failure Mechanisms

### 4.1. Failure Modes of Concrete Beams with Different Reinforcement

**Figure 7:**
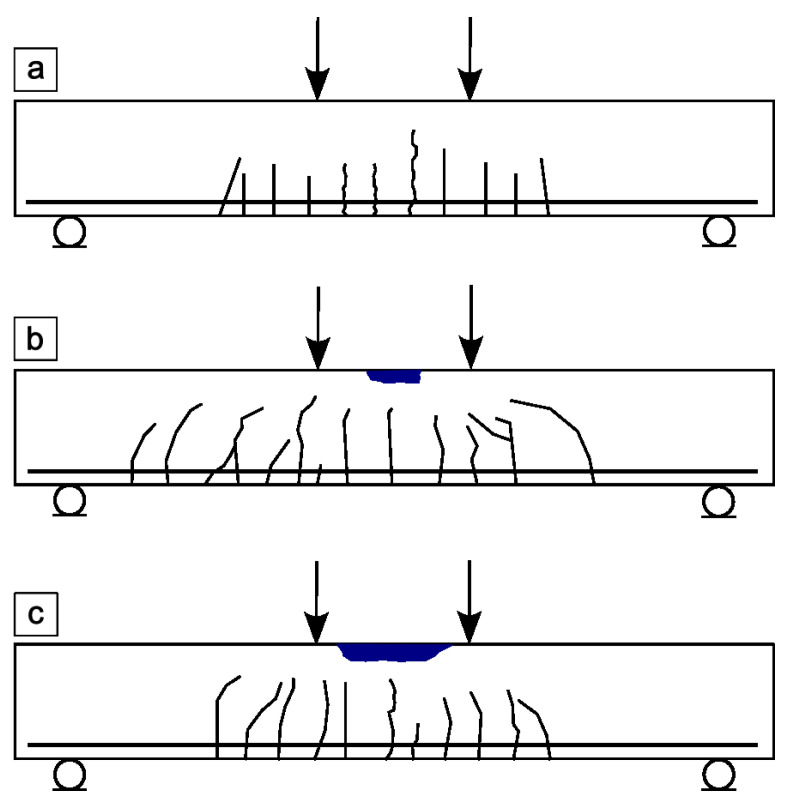
Schematic diagram of the failure of beams with different reinforcements: (**a**) steel bars; (**b**) composite bars; (**c**) hybrid bars.

The failure mechanism of a reinforced concrete beam with steel reinforcement, shown in Figure 7a, is continuous and predictable, which increases the safety of the structure. The process begins with the appearance of numerous small cracks in the tension zone, primarily in the central part of the span, where these are well-dispersed vertical cracks initiated from below. As the load increases, these cracks deepen and spread towards the supports, and their development is controlled by the main steel bars. After steel yields in the tension zone, the damage continues to spread, until the concrete in the compression zone crushes [89]. Due to the high ductility of the steel bars, the beam can withstand significant loads after cracking, maintaining its load-bearing capacity and signaling imminent failure through visible deformation, cracking sounds, and distinct displacements. In the case of UHPC, the crack network is more developed; the cracks are thinner but more numerous. Fibers in concrete can limit the width of the crack, improving the continuity of behavior and controlling crack propagation.

One of the key advantages of this type of failure mechanism is the predictability of the damage process. The gradual development of deformations and cracks allows effective monitoring of the technical condition of the structure, allowing early detection of irregularities [49,89,101]. Another advantage is the ductility of reinforced concrete elements, which exhibit the ability to undergo significant deformations before reaching the failure limit. This allows the structure to continue to function as a load-bearing structure even in the presence of severe damage [90,101,104]. Visible warning signs, such as cracks, deformations, or sounds accompanying cracking, are also notable, allowing users and technical services to take appropriate preventive measures [89,90]. Another significant advantage is the high efficiency of steel reinforcement, which effectively transfers tensile stresses, limiting crack development and increasing the operational safety of the structure [49,50,104]. Despite its numerous advantages, this type of failure mechanism is also associated with certain risks and limitations. One of the main threats is the crushing of the concrete in the compression zone, which can cause sudden and uncontrolled failure of the element [89,101,105]. In the case of insufficient transverse reinforcement, there is also the possibility of local shear failures, which can take on an unpredictable nature and result in a serious weakening of the load-bearing capacity of the structure [104]. Furthermore, this mechanism requires a precise design of both the main and transverse reinforcement layouts. An improper bar arrangement can disrupt the expected and favorable nature of failure, leading to premature failure [103,104].

Based on the analysis of studies available in the literature [49,50,101,102,103,104,105,111], it can be concluded that the failure mechanism of beams reinforced with composite bars, presented in Figure 7b, is most often brittle in nature. Composite bars, unlike traditional steel bars, do not exhibit the ability to yield, which results in a sudden and difficult-to-predict failure of the element after reaching the ultimate strength. In the case of GFRP-reinforced beams, the most frequently observed are the development of deep, concentrated cracks [49,50,101,103,104] dominant diagonal cracks and damage in the compression zone, such as concrete crushing or spalling of the concrete cover. GFRP has limited adhesion to concrete and low deformation capacity, which favors sudden failure without prior warning signals [104]. The lack of transverse reinforcement leads to rapid development of shear cracks. However, the use of carbon stirrups, especially in a fully closed configuration and with a close spacing, effectively controls the development of these cracks and improves overall beam performance. CFRP-reinforced beams, on the other hand, exhibit a more dispersed and controlled failure mechanism. Small, small-scale cracks appear on their surface, and the failure process itself usually involves crushing the concrete in the compression zone. This does not result in sudden concrete detachment or the formation of large cracks. Furthermore, the use of advanced materials such as UHPC [50] or ECC [106] significantly improves crack distribution and limits crack opening. However, the main disadvantage of composite bars, especially those reinforced with glass fiber, is their brittle nature of failure and the inability to redistribute stresses after the ultimate strength is exceeded. This requires the use of additional measures, such as steel fibers or stirrups, to improve structural performance.

The failure mechanism of the hybrid bar-reinforced beams, shown in Figure 7c, is characterized by a variety of damage scenarios that depend primarily on the material used in the outer layer of the bar and the type of core. The analyses conducted in [89,90,101] indicate that in all cases, there is no clear yielding phase or warning signs of failure, making the prediction of failure difficult. The failure process typically begins with the yielding of the steel core, followed by sudden failure of the outer composite layer. This leads to a sudden break in the bar’s continuity and ultimately to crushing of the concrete. In the case of fully composite bars with a glass fiber core, damage caused by material defects in the concrete occurs, which can lead to premature failure of the structure. The advantages of this failure mechanism include high load-bearing capacity until failure, resulting from the synergy of materials used in hybrid bars, corrosion resistance due to composite outer layers that effectively protect the steel core or are themselves durable (e.g., glass, carbon fibers, or basalt fibers), and lightweight construction, as composite bars are lighter than steel bars, which translates into a reduced component weight. However, the lack of a warning phase poses a serious risk to user safety. Furthermore, the behavior of the components is strongly dependent on the quality of the workmanship and material, and concrete defects can lead to premature failure [112]. The difficulties in detecting internal damage result from the fact that the outer coating of the composite can mask the damage to the core. Furthermore, the behavior of the bars varies depending on the type of fiber used. Basalt fibers cause thin, abrupt cracks [90], carbon fibers lead to a complete rupture of the cross-section [89], while glass fibers reduce the width and spacing of cracks [101].

### 4.2. Discussion

The analysis of the failure mechanisms presented for beams with different types of reinforcement, summarized in Table 6, shows that differences in the failure mechanism result primarily from the material properties of the bar and the interaction between the reinforcement and the concrete. Differences in the behavior of the structural element are influenced by the modulus of elasticity and deformability of the material, the bond between the bar and the concrete, the type of dominant stresses in the element and the effectiveness of the transverse reinforcement. Steel bars are characterized by the ability to undergo large deformations before failure, whereas carbon fiber-reinforced composite bars do not possess this ability. Furthermore, steel stirrups control crack development more effectively than composite stirrups, which increases the predictability of failure. Each type of reinforcement has its own application under different conditions. Steel reinforcement is optimal for structures where operational safety and evacuation are a priority, such as buildings or structures exposed to shocks and overloads. Composite reinforcement performs best in highly corrosive environments, such as waterfronts, treatment plants, and chemical industry facilities, where longevity and corrosion resistance are more important than warning signs of failure. Hybrid reinforcement, on the other hand, can be a good solution for structures that require a compromise between corrosion resistance and partial ductility, such as bridges and footbridges.

Future efforts should be made to improve the safety and predictability of failures in composite and hybrid reinforced structures. In the case of composite reinforcement, consideration could be given to developing methods for introducing artificial warning signals, such as strain sensors or fibers that change color when stress limits are reached. A better design of the transverse reinforcement would also be crucial to more effectively control the development of cracks. In hybrid reinforcement, the proportions of the steel core and composite cover should be optimized to extend the plastic phase and delay rapid failure. Furthermore, it is worthwhile to conduct research on the behavior of these structures under fatigue conditions, long-term loading, and dynamic loading. Furthermore, the development of numerical models that predict crack initiation and its development under various conditions could significantly improve the safety of the structures designed.

## 5. Analysis of Experimental Studies

### 5.1. Review of Experimental Studies on the Effects of Composite and Hybrid Bars

A summary of the research in selected publications is provided in Table 7. The analyzed research focused on beams made of ordinary concrete. At the same time, several researchers examined the differences between beams made of ordinary concrete, UHPC, and ECC, the impact of which on the results is discussed in the following chapter.

A study by Sun et al. [90] aimed to evaluate the effect of SBFCB reinforcement on the load-bearing capacity of flexural beams. Concrete with a compressive strength of approximately 39 MPa was used, and HRB400 steel bars and SBFCB bars, consisting of an inner steel bar core and an outer basalt fiber core with varying numbers of fiber bundles, were used for reinforcement. Two series of beams with different reinforcement configurations were prepared and tested in a four-point bending system. The first series analyzed the effect of reinforcement type, including steel bar-reinforced beams, hybrid beams (i.e., SBFCB and steel bars), and SBFCB beams. The second series investigated the effect of fiber content, including only SBFCB beams, while varying the number of fiber bundles in the composite bars. The study assessed the effect of the type of longitudinal reinforcement and the equivalent reinforcement ratio on the flexural load-bearing capacity of the beams. The tests carried out demonstrated that replacing traditional steel reinforcement with SBFCB hybrid reinforcement significantly improved the strength parameters of the tested beams. The beam reinforced only with SBFCB achieved the highest cracking force (F_cr_) and the ultimate force (F_u_), which were 69% and 83% higher, respectively, than the reinforced concrete beam. The hybrid beams, combining steel bars and SBFCB, demonstrated a gradual increase in load-bearing capacity with increasing hybrid bar ratio. The increase in critical and ultimate forces was clearly correlated with the SBFCB reinforcement ratio, confirming the beneficial effect of composite reinforcement on the load-bearing capacity of the elements. Detailed analysis indicated that the first vertical cracks appeared at loads close to the cracking forces, and their width increased with increasing load. It was observed that after exceeding the yield point of the steel and composite bars, the crack width increased to approximately 0.25–0.30 mm, with maximum cracks reaching up to 1.9 mm at the ultimate load. Furthermore, the results showed that increasing the longitudinal reinforcement ratio significantly improved the ultimate load capacity of the tested beams, both for steel and hybrid reinforcement.

In the study by Etman et al. [89], three types of hybrid bars were used, differing in steel core diameter (10 mm or 12 mm) and number of carbon fiber layers (2 or 3). Fourteen beams with identical dimensions (200 × 300 × 2000 mm) were tested, varying in terms of reinforcement type and concrete grade. All beams were heavily reinforced with stirrups to eliminate shear damage. The research program was divided into three groups. Group one consisted of six beams made of 30 MPa concrete, reinforced with steel bars or SCFCB. Group two contained four beams made of 40 MPa concrete, two reinforced with steel bars and two with SCFCB. Group three also included four beams, this time made of 50 MPa concrete and with reinforcement corresponding to group two. The study evaluated the flexural behavior of beams, analyzing the effect of reinforcement type and concrete grade on the load-bearing capacity and deformability of the members. A reinforced concrete beam served as the control beam. The introduction of SCFCBs to beam reinforcement significantly improved the mechanical performance of the tested beams. Specifically, the cracking force increased from 8% to 28% in the hybrid beams compared to the control beam. The increase in ultimate force was even more pronounced, ranging from 25% to 50%. The beam with more SCFCBs performed better than the beam with steel bars, indicating that a higher proportion of hybrid bars translates into higher load-bearing capacity. The beam reinforced solely with SCFCBs demonstrated the highest values of both cracking force and ultimate force, improving load-bearing capacity relative to the control beam by 37% and 70%, respectively. Failure mode analysis showed that all tested beams exhibited initial cracking in the bending zone, in the zone of maximum moment, i.e., in the center of the beam. Over time, cracks propagated towards the supports, becoming deeper and wider with increasing loading. Cracking loads were similar for beams with similar concrete and reinforcement properties. Increasing the concrete strength resulted in increases in cracking loads by up to 20–35%. Ultimate loads were also related to concrete strength, with SCFCB-reinforced beams achieving ultimate loads similar to reinforced concrete beams with the same concrete grade. The increase in concrete compressive strength translated into higher ultimate loads, confirming the increased load-bearing capacity of the members. The use of SCFCB reinforcement resulted in increased deflections after the first cracks appeared at the same load level, a consequence of the composite’s lower modulus of elasticity compared to steel. However, the ultimate deflection capacity was lower than that of steel beams due to the limited deformation capacity of FRP.

Abdel-Karim et al. [101] investigated RC beams reinforced with steel, GFRP, and hybrid steel–GFRP composite bars (SGFCBs), and evaluated the addition of polypropylene microfibers (PP) and macrofibers (MPP) at 1% and 2% by cement mass. Fourteen 2200 mm beams were tested in four-point bending: three controls with 3×Ø12 steel bars, two beams with 3×Ø12 GFRP, and nine SGFCB variants (typically 3×Ø14 baseline plus geometry/layout changes). Hybrid bars comprised 50% steel and 50% glass fibers with ribbed surfaces to improve bond. Results show that reinforcement type and PP additions govern performance. Steel and SGFCB beams exhibited higher load capacity and stiffness than GFRP, with yielding followed by concrete crushing. Over-reinforced cases failed by concrete crushing before yielding. As detailed, PP micro/macro-fibers enhance all three reinforcement cases: for steel (3#12, NC), F_cr_ rises from 22 to 29 kN (+32%) and F_u_ from 110 to 140 kN (+27%); for GFRP (3#12, NC), F_cr_ from 18 to 28 kN (+56%) and F_u_ from 96 to 113 kN (+18%); for SGFCB (3#14, NC baseline), 0.5% + 0.5% PP and 1% + 1% PP yield F_cr_ +50–60% and F_u_ +14–19%. Geometry also mattered and increased sections achieved higher capacities (e.g., SGFCB 3#14 up to 32/222 kN), while reduced sections markedly lowered strength (e.g., 28/80 kN).

Lu et al. [49] compared the behavior of GFRP-reinforced beams and steel bars under bending loads. The study used vinyl ester resin-coated GFRP and HRB400 steel bars of 12 mm diameter. The beams were made of concrete with a compressive strength of 50 MPa and 35 mm long, 0.5 mm diameter hooked steel fibers. Seven beams measuring 180 × 300 mm and 1800 mm long were prepared with single-layer and double-layer reinforcement and tested in four-point bending. Four of the beams were reinforced with a hybrid method combining GFRP and steel bars, one beam was reinforced only with GFRP, and the other two were reinforced only with steel bars. The study assessed the effect of GFRP and steel bars on beam strength and proposed a reinforcement ratio to evaluate hybrid-reinforced beams. Analysis of the test results indicated that the cracking and failure forces of the tested beams were clearly dependent on the type of reinforcement used and its configuration. The control beam had a higher cracking force than the glass fiber-reinforced beam, but the ultimate load-bearing capacity of the hybrid bar beams exceeded both the control sample and the GFRP beam. The authors emphasized that partially replacing the steel bars with GFRP in the hybrid beams resulted in a slight reduction in the cracking load but a simultaneous increase in the ultimate load-bearing capacity. The experimental results also showed that the failure mode differed depending on the reinforcement used. The GFRP beams failed mainly due to concrete crushing in the compression zone, without any bar fracture. However, the reinforced steel bars beams underwent a classical flexural failure. A comparison of the beams with and without steel fibers demonstrated clear benefits of using steel fibers, as the beams demonstrated a higher ultimate load-bearing capacity. In the case of hybrid reinforced beams, this increase was even more noticeable, which demonstrates the synergistic effect of concrete and the combination of GFRP and steel reinforcement.

In the study by Feng et al. [104], the shear capacity of reinforced concrete and GFRP beams was analyzed using different types of CFRP stirrup. Concrete class C30 was used, along with seven beams, each 1500 mm long and 150 × 250 mm in cross-section, with equivalent longitudinal reinforcement. In the case of reinforced concrete (RC) beams, the longitudinal and assembly reinforcement was made of HRB400 steel bars with diameters of 14 mm and 8 mm, respectively, while GFRP bars of the same diameter were used in the remaining beams. The stirrups in the RC beams were made of HPB300 steel bars with a diameter of 6 mm, while in the remaining beams, two types of CFRP strip stirrups were used. The purpose of the study was to determine the effect of the type and arrangement of reinforcement on shear capacity and failure mechanism. A 30 MPa control beam reinforced with steel bars and steel stirrups. The composite beam containing only glass bars without additional carbon stirrups achieved lower strength values, with F_cr_ of 20.8 kN and F_u_ of 31.4 kN, which are 82% and 60% of the control beam’s values, respectively. This indicates that the glass bars alone do not improve the structure’s load-bearing capacity; in fact, they reduce it. The introduction of carbon stirrups along with the glass bars significantly increased the load-bearing capacity of the composite beam. F_cr_ values increased from 93% to 113% of the control beam’s load-bearing capacity, while the ultimate load values increased from 69% to 106% of the control beam’s load-bearing capacity. The highest load-bearing capacity values were achieved with the densest carbon stirrup spacing of 6 mm. F_cr_ then increased by 29 kN, and Fu by 55.6 kN, exceeding the results of the control beam. The study observed that, as the load increased, numerous diagonal shear cracks appeared in two zones of the shear span. Of these, one crack developed rapidly and continuously towards the load points, leading to the formation of the main crack. In the composite beams, similar diagonal crack development was observed at an early stage, but additionally, cracking sounds were heard in the CFRP strips. The author notes that in the case of the beams with carbon stirrups, the longitudinal strips cracked first, followed by the transverse strips. During the study, the deformations of the stirrups were also measured. Before the concrete cracked, the deformations were small because the shear force was primarily transferred by the concrete. After the cracks appeared, the rate of growth of the stirrups’ deformations increased rapidly, indicating their significant contribution to the shear capacity. In the composite beams, the deformations of the CFRP strips did not reach the limit values, which means that they did not break during the test.

Liu et al. [112] analyzed the effect of hybrid bars, consisting of an FRP core embedded in an annular UHPC layer and an outer layer of FRP sheets coated with quartz sand, on the behavior of FRP-reinforced beams subjected to four-point bending. Nine beams with dimensions of 200 × 300 mm and lengths of 3100 mm were designed for the study. Two of these were conventional beams that served as control beams, while the remaining ones were reinforced with hybrid bars placed in compression zones or in both compression and tension zones. In the study, three types of hybrid bars with a total diameter of approximately 46 mm were used as reinforcement in compression and tension zones using ultra-high-performance concrete. The tests were performed on beams with different concrete parameters, spans, and reinforcement ratios, loaded using the four-point bending method. The study aimed to evaluate the strength and ductility of beams with different hybrid bar configurations. Increasing the amount of reinforcement in the tension zone by adding additional carbon rods leads to a significant increase in the initial stiffness of the element, which manifests itself in a higher cracking force. However, the effect of this treatment on the final load-bearing capacity of the structure is relatively small. In turn, the use of hybrid reinforcement in the compression zone, especially in the form of thick glass rods, significantly improves both the cracking force and the ultimate load. Properly selected reinforcement can also effectively compensate for lower concrete grades, allowing for very good load-bearing capacity despite poorer material properties. The best results were achieved for beams that combined thick glass reinforcement in the compression zone with three carbon rods in the tension zone. This configuration yielded the highest ultimate load-bearing force values among all tested specimens. However, not all hybrid systems proved to be effective. In some cases, despite the larger number of rods, the ultimate load-bearing capacity was lower than in the control beams. This may indicate suboptimal material selection or problems related to differences in stiffness. Ultimately, the study confirmed that the proper configuration and placement of the reinforcement is crucial to the efficiency of the structure.

Zhang et al. [113] tested seven FRP-reinforced beams with different configurations. One beam was reinforced with GFRP and served as a control beam, and the others had hybrid compression bars with different mechanical properties. All beams had the same dimensions of 200 × 300 mm and a length of 3100 mm. The hybrid bars were designed to exhibit different compressive behaviors, achieving both softening and hardening as a function of stress. The bars were manufactured by embedding a GFRP core in a UHPC-filled tube and wrapping it with FRP sheets with quartz sand for better adhesion. The control beam, designated as a composite beam, achieved a cracking force of 18 kN and an ultimate force of 248 kN. The introduction of hybrid bars into concrete, with a glass rod core of various diameters and an outer layer of glass fiber or carbon fiber, significantly improved the load-bearing capacity of the tested beams. The cracking force increased to as much as 24 kN, representing an increase of approximately 33% compared to the control beam. An even greater increase was observed in the ultimate force, which increased from approximately 32% to over 44% compared to the control beam. The best results were obtained for beams with reinforcement consisting of three 25 mm diameter glass rods and two hybrid rods with a glass fiber core and a glass fiber outer layer. The authors of the study noted four different failure modes that occurred during the tests. The control beam failed due to compressive crushing of the concrete on the beam’s top surface and spalling of the concrete cover, which caused shear fracture of the GFRP. This mode is characteristic of over-reinforced FRP beams. Unlike the control beam, beams reinforced with hybrid bars demonstrated the ability to continue to carry load even after the concrete on the beam’s top surface was crushed. This is due to the high strength of these bars. Although a momentary load drop was observed during concrete crushing, after the hybrid bars were activated, the load-bearing capacity began to increase again until ultimate failure, which could have occurred through compressive fracture of the hybrid bars or tensile fracture of the GFRP. Furthermore, the authors noted that the type of hybrid bars influenced the mode and nature of failure. Hybrid bars that exhibited softening behavior after reaching the peak resulted in lower load-bearing capacity and beam deflection, and their failure was characterized by compressive fracture of the hybrid bars. However, beams with hybrid bars that exhibited hardening behavior after reaching the peak demonstrated higher load-bearing capacity, and failure occurred through a sudden tensile fracture of the GFRP. The study also confirmed that increasing the GFRP tensile reinforcement ratio positively affected the stiffness and load-bearing capacity of the beam. However, the differences between beams with a larger number of GFRP bars were smaller, which the authors explained by the arrangement of the bars in several layers, which limits the effective use of reinforcement.

The study by Sun et al. [102] investigated beams reinforced with prestressed carbon fiber bars with different rib depths (0.2 mm and 0.6 mm) to assess their effect on structural behavior. Concrete with a compressive strength of 40.5 MPa was used, and some beams contained ultra-high-durability concrete (UHDC). CFRP was placed exclusively in the tensile zone of the beams as prestressed longitudinal reinforcement, while steel bars served as compression reinforcement and stirrups. Eight beams with a cross-section of 200 × 330 mm and a length of 2800 mm were tested for flexural resistance. The addition of UHDC to the beams improved the mechanical properties. For the beam with the same reinforcement as the control, the addition of UHDC increased F_cr_ by 13% and F_u_ by 14%. For samples with three 10 mm diameter bars and 0.2 mm ribs, F_cr_ increased by only 0.7%, while F_u_ increased by 15%. The largest increase in cracking force, thanks to UHDC, was observed in the sample with one 10 mm diameter bar and 0.6 mm ribs, where the increase was 20% for F_cr_ and 16% for F_u_. Increasing the number of reinforcing bars from one to three significantly increased both the cracking force and the maximum breaking force. For the 0.2 mm ribs without UHDC, F_cr_ increased by as much as 118%, and F_u_ by 154%. Even in samples with UHDC, the increases were significant, by 93% and 156%, respectively. Similar trends were observed at a 0.6 mm rib depth, where the increase in F_u_ exceeded 170%. Counterintuitively, increasing the rib depth from 0.2 mm to 0.6 mm did not produce positive results. For the same conditions, deeper ribs caused decreases in the F_cr_ and F_u_ values. For a single 10 mm diameter bar configuration without UHDC, the cracking force decreased by 18%, and the maximum breaking force by 11%. It can be assumed that the greater rib depth could have led to local concrete damage or impaired bonding between the bar and the matrix.

A study by Abadel [50] aimed to evaluate the effect of different concrete mixtures and the use of steel and composite bars on the load-bearing capacity of beams. Normal-density concrete was designed with a compressive strength of 30 MPa, and UHPC was designed with a compressive strength of 140 MPa, with the addition of steel microfibers that were 30 mm long and 0.5 mm in diameter at a volume of 1%. Longitudinal reinforcement consisted of steel bars with diameters of 8 and 10 mm and 10 mm diameter GFRP. Eight beams with a cross-section of 120 × 185 mm and a length of 1500 mm were manufactured with different combinations of steel and GFRP reinforcement and two types of concrete. Two beams served as controls: one with steel bars only, the other with GFRP only. The remaining beams were divided into two groups, each with a different concrete mix. The first group was made of normal concrete (NC), and the second group was made of UHPC. The tests conducted showed that the type of reinforcement and the concrete material used have a significant impact on the behavior of beams subjected to bending. Beams reinforced solely with GFRP exhibited significant concrete cracking, with the first signs of damage appearing at 10 kN, indicating limited initial stiffness and susceptibility to early cracking, but very good final properties. In beams combining steel and GFRP bars, an increase in F_cr_ and F_u_ was observed. The tests showed that, at initial loading, the steel bars transferred a greater proportion of the tensile forces, resulting in higher F_cr_ values. After the bars yielded, the forces were absorbed by the GFRP, ensuring high ultimate load-bearing capacity.

Li et al. [103] analyzed the effect of GFRP and HRB500 steel bars on the strength of lightweight ultra-high-performance fiber-reinforced concrete (LUHPC) beams with the addition of steel fibers 13 mm long and 0.22 mm in diameter. GFRP and steel bars with diameters of 12, 18 and 22 mm were used as longitudinal reinforcement. The test beams had dimensions of 150 × 300 mm, were 1800 mm long, and were tested using the four-point bending method on a universal testing machine. The study considered three levels of reinforcement ratio, thus determining the effect of the type and amount of reinforcement on the load-bearing capacity and the fracture mode of the LUHPC beams. The study showed that in composite and steel bar-reinforced beams, increasing the diameter of the reinforcement led to a significant increase in the load-bearing capacity of the members. In the case of GFRP, increasing the diameter resulted in an increase in the cracking force by more than 11% and a more than doubling of the maximum load-bearing capacity. A similar trend was observed in reinforced concrete beams, where the largest bars contributed to an increase in load-bearing capacity by nearly 186% compared to the control beam. The results confirmed a significant effect of reinforcement diameter on the behavior of the beams under load. Failure modes of the tested beams varied depending on the type of reinforcement. In the composite beams, the dominant failure mode was tensile fracture of the GFRP, leading to brittle failure. The study showed that increasing the reinforcement ratio significantly increased both cracking and failure loads. Crack analysis revealed that cracks in the composite beams developed faster and were wider, which was associated with a lower modulus of elasticity and poorer bonding between the GFRP and the concrete. In the reinforced concrete beams, cracks were more numerous but narrower, which is beneficial for the durability and aesthetics of the structure.

Xiang et al. [111] investigated the behavior of FRP-reinforced beams of various configurations, measuring 250 × 130 mm and 1700 mm long. UHPC, with the addition of 2% steel fibers, measuring 13 mm long and 0.2 mm in diameter, had a compressive strength of 159 MPa and a high modulus of elasticity. Four-point bending tests were performed to analyze the effect of reinforcement configuration on the load-bearing capacity and behavior of the beams. A control beam reinforced with carbon bars of 12, 14, and 18 mm diameters, supplemented with steel bars in the compression zone and stirrups, achieved a cracking force of 7.4 kN and a ultimate force of 85 kN, which were taken as reference values. In subsequent beams, the number and diameter of bars were varied, maintaining similar material conditions and dimensions. The highest F_cr_ among the tested beams was achieved by the 12 mm diameter GFRP-reinforced beam 2, reaching 7.5 kN, which is 101% of the reference beam. However, the ultimate load for this slab was lower, reaching 68 kN, or approximately 81% of the reference value. Cross-section dimensions also proved to be a significant factor influencing the slab’s load-bearing capacity. The beam with a reduced cross-sectional size of 250 × 100 mm had a significantly lower critical load of 4.7 kN and ultimate load of 57 kN, demonstrating that a smaller cross-section reduces the flexural load-bearing capacity. The authors noted that during the test, all beams failed due to concrete crushing in the compression zone, and in some cases, the FRP also cracked. A characteristic feature is that after the first crack occurred, numerous microcracks developed, but the stiffness of the samples did not decrease rapidly, which the authors attribute to the bridging effect of the UHPC fibers. This resulted in improved structural performance compared to traditional FRP-reinforced concretes, including a smaller stiffness decrease after cracking and controlled crack widths under live loads. The results confirmed that the fracture moments and deflections of beams of the same thickness were similar, indicating a minor effect of the FRP configuration on these parameters. However, the maximum crack widths under live loads remained well below the 0.5 mm limit, demonstrating the high effectiveness of UHPC in controlling crack growth. Furthermore, the authors noted that using smaller bar diameters with closer spacing and similar reinforcement ratios allows for higher bending moments and better energy dissipation, which translates into higher load-bearing capacity and durability. The effect of bar diameter is more noticeable at higher load levels, as the tensile UHPC fraction decreases, and the slippage between the FRP and concrete increases. Under such conditions, beams with smaller bar diameters achieve better results by reducing bar deformation and crack width, which highlights the importance of appropriate selection of the reinforcement system for the efficiency of the structure.

A study by Zhou et al. [105] aimed to evaluate the effect of the type of matrix and the type and configuration of longitudinal reinforcement on the flexural behavior of the beams. Nine beams with identical dimensions of 140 × 190 mm and a length of 1700 mm were prepared, seven of which were made of engineered cementitious composite (ECC) with the addition of polyvinyl alcohol (PVA) fibers, with a length of 12 mm, a diameter of 0.039 mm, and a volume of 2%, and two of which were made of plain concrete and used as control beams. The beams were reinforced with 12 mm long, 0.039 mm diameter PVA fibers with a 2% volume, longitudinal bars of 6, 10, and 13 mm diameters and varying amounts, with most designed with over-reinforcement to achieve a matrix crushing failure mode. The FRPs were sandblasted and anchored in sleeves. All beams were tested using a four-point loading test. The effects of reinforcement and matrix on the load-bearing capacity, fracture, and failure mechanism of the beams were analyzed. The control beam, made of concrete with a compressive strength of 40 MPa and reinforced with three 10 mm diameter steel bars, achieved a cracking force of 14.1 kN and a maximum ultimate force of 83.1 kN. The remaining beams were made of ECC and reinforced with carbon or glass bars, allowing for a comparison of the effects of both the type of reinforcement and the concrete properties on the load-bearing capacity and behavior of the structure. The use of carbon reinforcement significantly increased the load-bearing capacity of the beams. The beam with three 10 mm diameter carbon bars, made of ECC, achieved a load-bearing capacity of 151.8 kN, which was 182% of the value of the control beam. Even higher results were achieved with higher reinforcement ratios, such as five 10 mm diameter bars or three 13 mm diameter bars, where the load-bearing capacity reached up to 215% of the reference beam. Even with the same concrete grade, the use of carbon bars provided a clear advantage, increasing the load-bearing capacity by 152% compared to the control beam. The glass bars also improved the load-bearing capacity, although to a lesser extent. The beam with three 10 mm diameter ECC bars achieved F_u_ = 103.2 kN, which is 24% higher than the control beam. For the same reinforcement configuration in 40 MPa concrete, the load-bearing capacity was 89.8 kN, which is 8% higher than the control beam.

Wang et al. [106] investigated the properties of longitudinal bars and stirrups made of GFRP and HRB400 steel. Plain concrete and ECC with polyethylene (PE) fibers, which improve crack resistance, were used in the tests. In the study, seven GFRP-reinforced beams were prepared, including five GFRP beams and two with hybrid bars that combine GFRP and steel. All beams had the same dimensions: 170 × 340 mm and a length of 2200 mm. The beams were subjected to four-point bending tests. The study aimed to evaluate the effect of the type and amount of reinforcement on the load-bearing capacity of the beams. The control beam was made of fiberless composite concrete and had a cracking force of 32.7 kN and a failure force of 261.9 kN. The addition of ECC reduced F_cr_ but improved F_u_. The beam with ECC and PE added had an F_cr_ of approximately 19.6 kN, which was approximately 60% of the control beam’s value, while the maximum force F_u_ increased by approximately 7%. Increasing the amount of reinforcement in the form of 20 mm diameter bars resulted in an increase in both the cracking and ultimate forces. Beams with three and four bars showed up to 122% and over 130% of the F_cr_ values, respectively, compared to the control beam. The highest load-bearing capacities were achieved in beams using glass and steel bars with stirrups. In these samples, the F_cr_ reached up to 50.1 kN, which was over 150% of the control value, and the F_cr_ reached 376 kN, which was over 140% of the maximum force of the control beam. Hybrid beams with ECC added also performed very well, with a significant increase in the maximum force F_u_ to 382.7 kN, although the F_cr_ value was lower than in the hybrid without ECC. The failure modes of beams made of glass fiber-reinforced concrete and ECC are similar to those of beams reinforced solely with glass fiber. Increasing the reinforcement ratio translates into improved load-bearing capacity and stiffness, although it also causes greater deflection at peak load. Furthermore, the use of hybrid reinforcement with steel bars allows for better crack propagation control and increases structural ductility, as evidenced by the higher ductility of the hybrid specimens.

Erfan et al. [120] conducted tests on seven wide high-performance concrete (HPC) beams to evaluate the effect of different types of reinforcement and the addition of steel fibers on the shear strength. The beams were made from three types of concrete mixtures: without fibers and with 1.5% and 3% steel fibers, each 50 mm long, while maintaining the same dimensions and longitudinal reinforcement. The research program was divided into three groups. The first group included two HPC beams without steel fibers, differing in the spacing of their 8 mm diameter steel stirrups, 125 mm and 200 mm. The second group consisted of two HPC beams with 1.5% and 3% fiber additions, reinforced with steel stirrups at a constant spacing of 200 mm. The third group consisted of three beams: one made of concrete without steel fibers reinforced with GFRP stirrups; the other made of concrete with 1.5% steel fibers and GFRP stirrups; and the third made of concrete without fibers reinforced with a hybrid system consisting of external steel stirrups and internal GFRP stirrups. GFRP stirrups were used due to their high tensile strength. The goal was to compare the effectiveness of different reinforcement arrangements and determine the effect of steel fibers on the mechanical properties of the beams. The results showed that multiple factors influence the failure mode and crack propagation, with the crack-free compression zone playing a key role, directing and limiting the development of diagonal cracks. Failure occurred only when critical shear cracks extended through the compression zone or the compressive strength of the concrete was exceeded. This explains why the high-performance concrete effectively resisted the shear force and why the control beams withstood high ultimate loads. Increasing the density of steel stirrups from 20 cm to 12.5 cm resulted in a slight increase in load-bearing capacity. Critical load increased by approximately 5% and maximum load-bearing capacity by approximately 4%. Adding steel fibers at 1.5% and 3% to 72 MPa concrete and using steel stirrups every 20 cm significantly improved the results. Crack strength increased by approximately 10% and 13%, respectively, and ultimate load by 9% and 24%. Glass fiber stirrups used in composite concrete demonstrated better performance than steel stirrups. Crack strength was approximately 19% higher, and ultimate load was 9% higher. The combination of steel and glass stirrups in the hybrid concrete specimen yielded the best results compared to the specimens without fiber addition, with crack strength increased by 53% and ultimate load by almost 30%. Tests showed that the beams were initially crack-free until peak stress was reached, after which vertical bending cracks began to form in the lower tension zone. Later, shear cracks moved toward the supports, and diagonal cracks appeared in the shear zone, which gradually grew with increasing load. Changes in the stirrup spacing, steel fiber content, and stirrup material type influenced the rate of diagonal crack propagation. Reducing the stirrup spacing from 200 mm to 125 mm increased shear capacity and delayed crack initiation. Adding steel fibers increased shear strength, improved ductility, and reduced crack width through crack bridging. Increasing the volumetric steel fiber content further improved mechanical properties and shifted the failure mode toward a more ductile failure. Replacing the steel stirrups with GFRB stirrups improved shear strength, but the failure mode became semi-brittle or brittle, resulting in a rapid and less favorable failure. However, using a hybrid system consisting of GFRB and steel stirrups and adding steel fibers to the concrete mix effectively improved the ductility and strength of the beams, combining the advantages of both materials.

### 5.2. Comparison of Research Results

#### 5.2.1. Structural Elements Reinforced with Composite Bars

Table 8 summarizes the properties of the beams reinforced with various composite bar systems, taking into account the fiber used and the concrete grade. The purpose of the analysis was to determine the effectiveness of reinforcement by comparing the type and amount of reinforcement with the corresponding values of the cracking and failure forces. In the case of CFRP-reinforced beams [102], the use of a single 10 mm diameter bar allowed for an F_u_ of 131 kN. The absence of fiber in the concrete in the beam in question allowed for a direct correlation between the load-bearing capacity and the type of reinforcement. The cracking force in this case was 55 kN, practically the same as the average achieved by the reinforced beams with steel bars. However, the failure force was approximately 78% of the average F_u_ for reinforced concrete beams. This is a very good result considering the significantly lower amount of reinforcement used. This confirms the high effectiveness of CFRP as a reinforcing material, which, with a lower volume fraction, can provide performance parameters comparable to steel. In contrast, GFRP-reinforced beams demonstrated significantly lower cracking and failure forces. For the beam with three glass fiber-reinforced bars, F_cr_ = 10 kN and F_u_ = 84 kN [50] were obtained, which is 50% of the failure force of the reinforced concrete beam and only about 18% of the cracking force. Another beam reinforced with three 12 mm diameter bars [101] achieved F_cr_ = 18 kN and F_u_ = 96 kN. These results confirm that GFRP, although cheaper and corrosion-resistant, has a lower modulus of elasticity and tensile strength than CFRP and steel bars. This results in lower beam stiffness and earlier cracking, as well as lower ultimate load-bearing capacity with typical reinforcement ratios. Increasing the number and diameter of GFRP bars can significantly improve beam load-bearing capacity. An example is a beam in which reinforcement consisting of three 25 mm diameter bars and two 12 mm diameter bars was used, with a concrete compressive strength of 55 MPa. In this case, the ultimate load reached 248 kN, which is higher than the average for beams reinforced with steel bars [113]. However, it should be noted that this was achieved at the cost of significantly increasing the amount of reinforcement, which may not be economically or structurally effective in standard solutions. Furthermore, the increased concrete grade may also have influenced the obtained results. In a beam in which two 14 mm diameter GFRP bars and additional 20 mm diameter carbon fiber stirrups were used, spaced 9 mm apart [104]. In this case, the cracking load reached 24 kN, which was higher than in the other GFRP beams, yet the ultimate load was only 36 kN. This may suggest that the use of additional transverse reinforcement has a beneficial effect on reducing cracking, but its effect on the ultimate load-bearing capacity was limited in this case.

**Figure 8:**
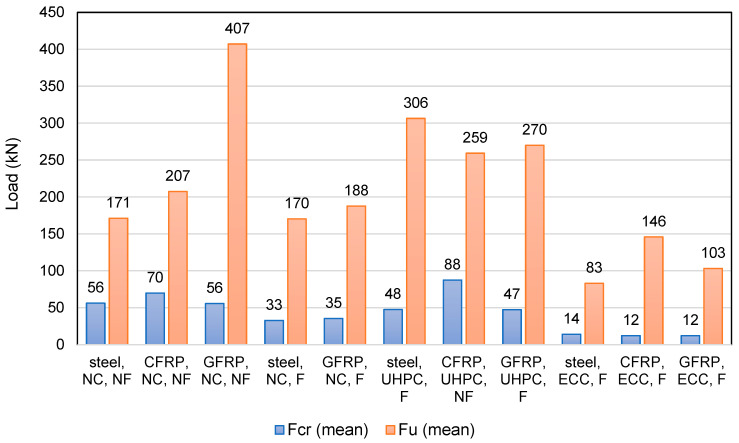
Comparison of cracking and ultimate loads for different reinforcements; (Note: F—with fibers, NF—without fibers).

The comparative analysis, the scope of which concerned the results of tests on beams made of ordinary concrete, presented in Figure 8, showed that the main factor influencing the load-bearing capacity of the beams was not only the concrete grade, but primarily the type and amount of reinforcement used. CFRP was shown to be significantly more structurally efficient, allowing higher values of destructive forces to be achieved with a smaller number and diameter of bars. However, GFRP bars, although advantageous in terms of chemical resistance and cost, require larger cross-sections to ensure comparable load-bearing capacity. The use of composite reinforcement in beams without the addition of fibers reveals significant differences in the effectiveness of individual reinforcing materials. CFRP demonstrates a clear advantage over GFRP in terms of load-bearing capacity and crack resistance, especially with limited reinforcement. However, a properly selected amount of GFRP can also provide high load-bearing capacity, albeit at the cost of higher material consumption. For designers, this means the need to carefully select the type of composite reinforcement, taking into account both its material properties and the specific behavior of the structural element.

#### 5.2.2. Structural Elements Reinforced with Hybrid Bars

Table 9 summarizes the properties of beams reinforced with various hybrid bar systems, with a steel core and an outer shell made of various types of fibers: basalt, carbon, and glass, taking into account the class of concrete, which covers the scope of test results for conventional concrete beams and the amount of reinforcement. Particular attention was paid to the analysis of cracking and destructive forces.

**Figure 9:**
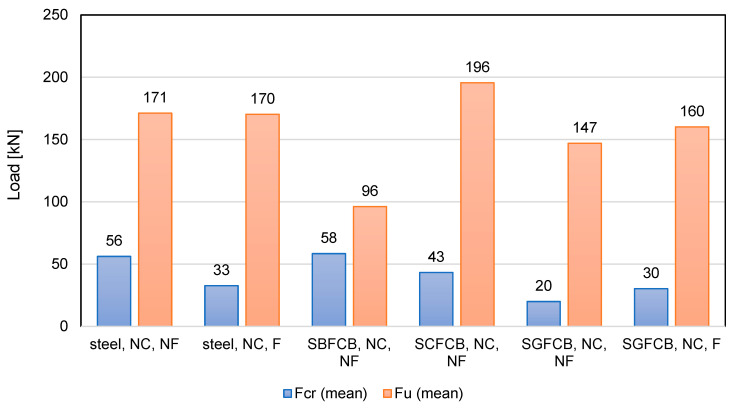
Comparison of cracking and ultimate loads for different reinforcements.

The beams reinforced by SBFCB [90] were made of C30 concrete. The number of bars used ranged from two to three, and their diameters ranged from 10.8 to 12.1 mm. The F_u_ values obtained varied from 64 to 203 kN, with the highest value for the beam with three bars of 11.5 mm diameter. The lowest values occurred in beams with less reinforcement. The average cracking force for the SBFCB-reinforced beams was approximately 48 kN, and the average ultimate force was approximately 91 kN. Compared to the reinforced beams with steel bars, this represents a decrease in F_cr_ of approximately 14% and a decrease in F_u_ of over 45%. These results indicate that the hybrid bars with a basalt coating are characterized by lower load-bearing capacity and lower stiffness, which may be due to weaker adhesion of the coating to the concrete or the lower stiffness of the coating itself compared to the steel bars. SCFCB-reinforced beams [89] demonstrated the best load-bearing capacity. Bars were used in pairs of two, with diameters ranging from 10.5 to 12.8 mm, and the beams were made of concrete grades C30, C40, and C50. Ultimate load values ranged from 93 to 288 kN, and the significant increase in load-bearing capacity was correlated with both the increase in concrete grade and bar diameter. The best results were obtained with reinforcement using two 12.8 mm diameter bars in 55 MPa concrete. The average cracking force for SCFCB-reinforced beams was approximately 45 kN, and the ultimate load was approximately 185 kN, meaning that F_u_ was approximately 10% higher than for reinforced concrete beams, even though F_cr_ was slightly lower. These results confirm that SCFCBs are the most effective alternative to traditional steel bar reinforcement among the types analyzed. In the case of SGFCB-reinforced beams [101], where the concrete had a constant compressive strength of 30 MPa, two to four 14 mm diameter bars were used. In some beams, a mixture of polypropylene fibers, 18 mm long and 0.03 mm in diameter, and macro-polypropylene fibers, 65 mm long and 0.5 mm in diameter, were also added. The ultimate forces ranged from 80 to 185 kN, with higher values achieved in beams with more bars and fiber additions. The beam with four bars and fiber additions achieved F_u_ = 185 kN, while the beams without additions and with fewer bars achieved lower values. The average cracking force for SGFCB-reinforced beams was approximately 28 kN, and the ultimate force was 147 kN, which means a decrease in F_cr_ by approximately 50% and F_u_ by approximately 12% compared to the reinforced concrete beams. Although the ultimate strength was relatively similar to that of steel-reinforced beams, the significantly lower initial stiffness can negatively impact the serviceability of the structure, for example, through increased deflection. However, the use of polypropylene fiber additives and a larger number of rebars improved the load-bearing capacity of SGFCB beams, indicating the potential for optimization of this type of reinforcement. A comparison of all three types of rebars, shown in Figure 9, shows that SCFCBs demonstrated the highest efficiency in terms of both cracking strength and ultimate strength. SGFCBs achieved moderate results, but their load-bearing capacity can be improved by increasing the amount of reinforcement and using PP fiber additives. SBFCBs proved to be the least effective, and their results varied, which may be due to lower stiffness or weaker bonding to concrete. Therefore, the type of fiber coating, the number and diameter of rebars, and the concrete grade all influence the load-bearing capacity of the beams. Selecting the appropriate type of hybrid rebar and optimizing its parameters are crucial for achieving high load-bearing capacity of structural elements.

#### 5.2.3. Structural Elements Reinforced with Hybrid Bars and Composite Bars

Table 10 and Table 11 summarize the properties of the reinforced beams with various hybrid and composite bar systems, taking into account the fiber used and the concrete class. Analysis of test results for concrete beams with a compressive strength of 30–50 MPa without fiber additions, obtained with three reinforcement systems: hybrid bars with a steel core and carbon fiber cover, classic composite bars reinforced with carbon fiber, and hybrid bars composed of a GFRP or CFRP core, a UHPC concrete layer, and an outer layer of carbon or glass fiber showed that the best results in terms of F_u_ and F_cr_ were achieved by SCFCB-reinforced beams. Due to the steel core, these bars ensure a very good interaction with the concrete and high ductility, resulting in a higher load-bearing capacity and stiffness with relatively little reinforcement. The tests recorded F_u_ of up to 288 kN and high cracking force values of up to 52 kN [89]. As the concrete strength and diameter of the bar increased, both the cracking force and the ultimate force were observed. In comparison, beams reinforced with CFRP composite bars achieved significantly lower ultimate forces of 126 kN [105], despite the use of a larger number of bars. This is due to the brittle nature of the material and its lower adhesion to concrete, leading to poorer interaction in the near-surface zone and lower stiffness. Despite the very high tensile strength of carbon fibers, their limited ductility limits the potential of CFRP bars in structures where continued service after the elastic limit is essential. In contrast, GFRP-UHPC-GFRP hybrid bars performed better than CFRP, but this required significantly more reinforcement [112]. This effect can be attributed to the use of a UHPC layer inside the bar, which improves stiffness and the adhesion of the reinforcement to the surrounding concrete, while the external composite layers provide durability and resistance to environmental factors. The production of these bars is technologically complex, which may limit their use in specialized structures. GFRP-reinforced beams were characterized by the lowest values of load-bearing capacity. In most cases, the ultimate load ranged from 31 to 96 kN [101,104,105]. SGFCB-reinforced beams achieved significantly higher ultimate load values, even with the same concrete grade. For beams with three 14 mm diameter SGFCBs, the ultimate load ranged from 147 to 175 kN [101], and with the number of bars increased to four, it reached up to 185 kN. Even better results were achieved with SGFCB + GFRP reinforcement (two 14 mm bars + three 12 mm bars), where the ultimate load reached 195 kN. The highest ultimate load values were achieved by beams reinforced with advanced GFRP-UHPC-GFRP. These beams achieved ultimate loads of up to 359 kN [113]. The mere presence of the UHPC GFRP layer allowed for an F_u_ greater than 248 kN, while the addition of an external glass fiber layer further increased strength while maintaining high corrosion resistance. It should be noted that the cracking force in these beams was not significantly higher than in the SGFCB beams, suggesting that their exceptional load-bearing capacity is primarily due to the synergistic effect of the entire hybrid reinforcement system and the high stiffness of the UHPC cover.

### 5.3. Discussion

Steel reinforcement has long been the primary reinforcement material for reinforced concrete structures. This is due to its applications, such as high initial stiffness, good compatibility with concrete, ductility, and predictable behavior after exceeding the yield point. Based on the literature reviewed, steel bar-reinforced beams achieve a cracking force of 56 kN and a failure force of 168 kN. These values provide a benchmark for alternative types of reinforcement.

Compared to steel bars, classic composite bars such as CFRP and GFRP are characterized by significantly lower initial stiffness and lack of ductility. Despite their very high tensile strength, CFRP exhibits brittle failure and poor adhesion to concrete, which limits their use in structures that require continued operation after cracking [105]. GFRP-reinforced beams are characterized by low ultimate strength in the range of 31 to 113 kN and cracking strength of 10 to 29 kN, which makes them suitable primarily for lightweight structures operating in aggressive environments [101,104,105]. Beams reinforced with hybrid bars, especially those containing a steel core, achieve significantly better mechanical parameters. They achieve average F_cr_ values of 46.5 kN and F_u_ values of 69.4 kN [90]. Although these values are lower than for reinforced concrete beams, the use of a larger number of bars allows for load-bearing capacities comparable to or even higher than those achieved with steel reinforcement. Their main advantage is higher corrosion resistance and lower weight, which can be beneficial in buildings exposed to aggressive environments. SGFCBs achieve even better results. The beams with this reinforcement achieved failure forces in the range of 131 to 195 kN and cracking forces of 20 to 42 kN, which means that they can even exceed the load-bearing capacity of the reinforced concrete beams while ensuring greater durability [101]. In particular, in configurations with a larger number of bars, the failure forces reached up to 195 kN. Similarly, SCFCBs demonstrate high ductility and very good interaction with concrete. The beams with this reinforcement achieved average F_u_ values of 195 kN and F_cr_ values of approximately 44.5 kN [89]. The presence of a steel reinforcement core provides the desired ductility, while the outer carbon fiber layer provides high corrosion resistance. This makes SCFCBs an attractive solution for bridges and engineering structures exposed to aggressive environmental factors. Among the solutions analyzed, the highest load-bearing capacity is achieved by beams reinforced with advanced GFRP-UHPC-GFRP hybrid bars. Due to the synergy of the composite core, the UHPC layer and the outer glass fiber layers, these beams achieve ultimate forces ranging from 243 to even 359 kN [113]. Although their cracking strength is lower than that of steel bars, their high ultimate strength and good resistance to environmental factors make them exceptionally effective in structures with high loads and durability requirements, such as strategic facilities, long-span bridges and critical infrastructure [112,113].

**Figure 10:**
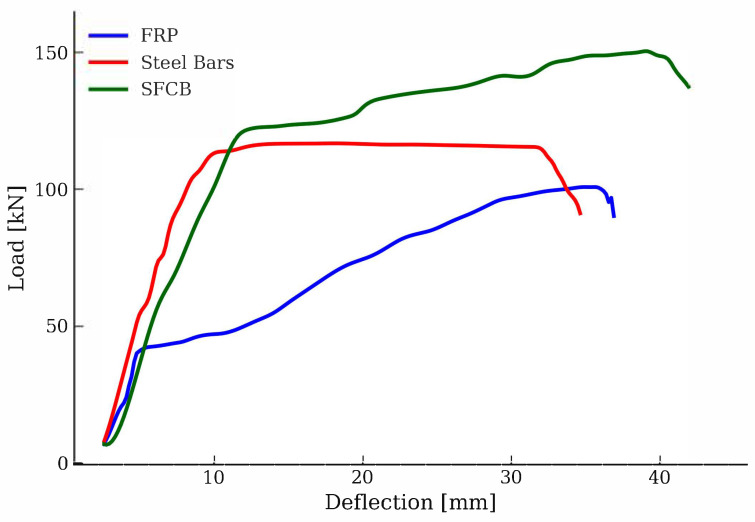
Load-deflection behavior of beams with different reinforcements.

All of these beams exhibit initial stiffness, which means that initially the load increases elastically with small deflections, as shown in Figure 10. However, there are significant differences in behavior depending on the type of reinforcement. Once the ultimate load is reached, the FRP-reinforced beam fails rapidly without prior warning. This is because FRP composites are characterized by low ductility and are brittle materials, which means they do not provide reserve load capacity after reaching their maximum. Steel bars exhibit higher ductility. After reaching their yield point, the steel-reinforced beam can still support the load for some time, giving it the opportunity to react before total failure. Hybrid reinforcement provides high ductility due to the steel core. After reaching maximum load, the SFCB exhibits a slow decrease in load capacity, ensuring the structure remains safe even after the maximum load is exceeded. Steel reinforcement remains a well-balanced solution in terms of stiffness, ductility, and cost. However, modern hybrid reinforcements, especially SCFCB and SGFCB, have a great potential to replace them in structures exposed to aggressive environments. Despite their high cost and production complexity, GFRP-UHPC-GFRP can be used in specialized structures requiring the highest load-bearing capacity and durability. Future research should further analyze the long-term behavior of hybrid reinforcement, particularly in terms of fatigue, the effects of high and low temperatures, and dynamic interactions. Comprehensive studies on the behavior of these reinforcements in exceptional situations, such as earthquakes, fires, or impacts, are also lacking. Life cycle assessment (LCA) studies are also crucial as they determine the cost-effectiveness of implementing new materials throughout the life cycle of the structure. The standardization of design and standardization methods for hybrid reinforcement is also needed, which will enable their broader application in engineering practice.

### 5.4. The Influence of Other Construction Materials on the Load-Bearing Capacity of Beams Reinforced with Various Types of Bar

Based on the analysis of the data presented in Table 7, the influence of UHPC, UHDC, ECC and fibers on the values of cracking forces and the ultimate forces of beams reinforced with composite and hybrid bars was examined.

Among the additives analyzed, UHDC, used in SCFCB beams, demonstrated the most beneficial effect, as indicated by the data summarized in [102]. Compared to beams with identical reinforcement but without UHDC, a noticeable increase in cracking force was observed by 15–20% and in ultimate force by approximately 14–15%. A similar increase was observed for bars with deeper ribs (0.6 mm), where F_cr_ increased from 45 kN to 54 kN, and F_u_ from 117 kN to 135 kN. With higher reinforcement ratios, the addition of UHDC had a smaller effect on the cracking force, but still led to an increase in ultimate force by approximately 15%, from 333 kN to 382 kN. This indicates that UHDC is particularly effective with less reinforcement, improving both initial stiffness and ultimate load-bearing capacity. A different situation was observed when ECC with PVA fibers was used in CFRP-reinforced beams, as illustrated by data from [105]. Despite the use of a highly ductile material, the obtained F_cr_ values remained low regardless of the number of bars. The ultimate load, although it increased with increasing reinforcement, did not exceed the levels achieved by beams reinforced with hybrid bars. The lack of significant improvement may be due to the limited adhesion of CFRP to concrete and its brittle failure mode. Despite its high tensile strength, CFRP does not provide plastic behavior beyond its elastic limit, thus preventing the full potential of ductile materials such as ECC from being fully utilized. The choice of reinforcement additive and type should therefore be closely related to the design requirements, particularly whether the priority is crack resistance and initial stiffness or maximum load-bearing capacity and long-term durability.

### 5.5. Durability and Long-Term Performance

Meeting contemporary durability and reliability demands in RC structures requires evidence beyond short-term mechanics. Two complementary studies address this under cyclic loading and aggressive exposure [121,122]. In [121], fourteen RC beams (1850 mm; 100 × 200 mm) with HRB 400 steel were strengthened using a 0.23 mm external CFRP laminate (E = 230 GPa). Two configurations were examined: unstressed and prestressed to 10% of CFRP tensile strength applied without altering the concrete cross-section to isolate strengthening effects. The program focused on high-cycle fatigue without direct environmental aging. Results showed a three-stage crack evolution: initiation, stable growth and sudden failure. Prestressing effectively limited crack propagation and shielded the steel from overload, enhancing fatigue resistance. Although creep was not directly tested, strain observations indicate indirect crack control via reduced steel deformation under prestress.

In [122], composite reinforcing bars with an 8 mm steel core encased in a pultruded GFRP shell (epoxy–vinyl ester) were evaluated using 200 × 200 × 200 mm concrete blocks with 5d anchorage and overall bar diameters of 12 and 16 mm. Long-term exposure in artificial seawater at 23 °C, 40 °C, and 60 °C for 30, 180, and 360 days targeted bond strength retention at the SFCB–concrete interface. SEM revealed matrix hydrolysis in the GFRP shell, leading to fiber detachment and microcracking. Bond retention decreased with time and temperature. Contributing factors included thermal-expansion mismatch, interface porosity, and chloride diffusion. While fatigue testing was not performed, the observed failure modes of bar pull-out or non-slip fracture suggest vulnerability to brittle response under repeated low-cycle/seismic demands.

Together, refs. [121,122] highlight the central role of the reinforcement–concrete interface in durability. In [121], the interface is effectively protected by the external CFRP laminate, delaying damage, whereas in [122], the aged SFCB–concrete interface becomes the weak link under marine exposure. Material selection, bar geometry (core diameter, shell thickness), and application technology directly affect long-term performance. Notably, prestressed CFRP can delay failure, and thicker GFRP shells better protect the steel core from corrosion and bond loss.

### 5.6. Standard Guidelines

Due to the different mechanical properties of FRP, such as low modulus of elasticity and lack of plastic behavior, the design of structural elements with this type of reinforcement requires a new approach. Compared to steel bars, FRP-reinforced elements are characterized by greater deflections and wider cracks in the service state, making these aspects crucial in the design process [101,111]. In response to these challenges, numerous technical organizations have developed design standards and guidelines that address the specific characteristics of composite reinforcement. Select commonly used design standards, their approach to serviceability and the resulting limitations and recommendations are discussed below. The ACI 440.1R-15 standard [18,105,123] and other international design guidelines, such as CAN/CSA S806-12 [124], JSCE [125] and GB 50608–2020 [126], emphasize that the key design criteria for FRP-reinforced composite beams are control of deflection and crack width. This is directly related to the low modulus of elasticity of FRP, which results in greater deformation in the service state compared to similar steel-reinforced elements [105,111]. These standards indicate that when designing FRP-reinforced beams, the first priority should be to ensure that the serviceability limit state requirements are met, often at the expense of fully utilizing the strength of the material. A high reinforcement ratio can reduce deflection and crack width, but leads to inefficient utilization of the FRP strength [111]. Additionally, design standards, such as ACI 440 [123], introduce the so-called Environmental reduction factors, which reduce the design strength of FRP bars depending on operating conditions such as humidity, temperature and chemical aggressiveness of the environment. Another significant factor limiting allowable stresses is creep rupture, particularly relevant for GFRP and AFRP bars, which exhibit relatively low fatigue strength. As a result, the standards impose additional constraints on long-term stress levels, which impact the design of elements with composite reinforcement in terms of serviceability and durability. ACI 318 [127,128] and ACI 440.1R-15 [18,123] present specific equations for predicting crack widths: the former for steel reinforcement and the latter for flexural elements with FRP bars. These standards also include additional factors that explain the weakened bond between concrete and FRP, as well as the linear behavior of composite bars up to failure [101]. Studies have shown that FRP-reinforced structural elements exhibit wider cracks and greater deflections than steel bar-reinforced beams for the same cross-section and reinforcement configuration [101,105]. Design standards therefore consider not only the strength of the materials, but also their bonding properties, slippage, and load transfer efficiency at the joints [129]. Furthermore, JSCE and CSA S806-12 [124,125,130] in their guidelines for the design of FRP reinforcement emphasize the importance of factors such as anchor length, fiber type, roughness of the bar surface, the presence of transverse reinforcement, and concrete strength. Ignoring these parameters can lead to significant errors in the prediction of bond strength [104,129]. In the context of shear, codes such as ACI 440.1R-15, CSA S806-12, GB 50608-2020 and BISE 1999 [123,124,126,131] base their calculation models on the classical truss model, but impose strict limits on the effective stress in FRP stirrups to reduce the risk of brittle failure. Limiting the stress in stirrups to 40% of the ultimate tensile strength or 0.4% of the elastic modulus is intended to improve the safety of the members [104]. Of the codes discussed above, ACI 440.1R-15 [123] demonstrated the closest agreement with the test results and is considered the more realistic of the codes analyzed [129]. The above analysis indicates that these design codes explicitly consider deflections and crack widths as the main design criteria for FRP-reinforced members, which distinguishes them from classical design methods for reinforced concrete elements. Although guidelines such as ACI 440.1R-15, CSA S806-12, and JSCE [123,124,125] provide an important basis for the design of FRP structures, further development of these documents is necessary to account for more factors influencing actual structural behavior. In particular, further research on bonding, shear, and high-temperature operation is needed to improve the reliability and optimality of composite reinforcement solutions.

## 6. Conclusions and Development Prospects

### 6.1. Summary of Key Findings

**Figure 11:**
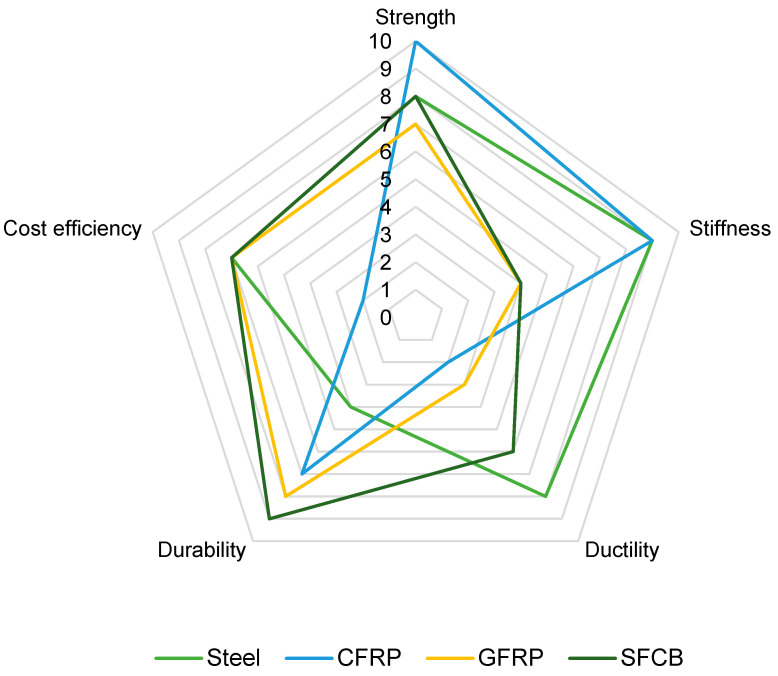
Radar chart of normalized scores (0–10, min–max): strength (mean f_u_), stiffness (mean E), ductility (post-peak softening/energy or curvature-ductility index), durability (corrosion/bond retention), and cost efficiency (relative cost per unit tensile capacity). The figure provides comparative profiles, not raw test values.

This article systematically reviews the literature on the effect of composite rebar on the mechanical properties of structural elements. A classification was made based on the type of reinforcement, concrete, and scope of research, with a particular emphasis on strength parameters, durability, and failure mechanisms. The results presented in Figure 11 summarizes five criteria on a 0–10 normalized scale (min–max): strength (mean f_u_ from Table 4), stiffness (mean E), ductility (post-peak softening/energy or curvature-ductility index from Figure 8 and Figure 9), durability (evidence on corrosion/bond retention), and cost efficiency (relative material cost per unit tensile capacity). The radar shows comparative profiles rather than raw values. A larger polygon area indicates a more favorable overall balance. In general, steel provides the highest ductility and cost efficiency but lower durability. CFRP excels in strength and stiffness with limited ductility. GFRP offers moderate performance with better economy. SFCB delivers a balanced profile by combining FRP-type durability with steel-core ductility. The purpose of the review was to present the current state of knowledge, indicate directions for future research, and support the development and application of composite technology in construction. Based on the analysis of the properties and behavior of different reinforcement types, the following points are most relevant for engineering practice:

1. Reinforcement selection must consider load, environment, durability, and budget. Steel remains indispensable in conventional structures for its predictable performance and safety margin. FRP is advantageous in aggressive environments, while hybrids provide a balanced option in intermediate cases.
2. CFRP offers high strength and corrosion resistance, suited for bridges and chemically aggressive exposures. Its brittleness and high cost restrict use to high-performance projects.
3. GFRP provides a cost–durability balance, useful in road and bridge infrastructure under variable climates, but limited ductility and chemical degradation require careful detailing.
4. Hybrids (SFCB/SCFCB/SGFCB) combine steel ductility with FRP durability, giving more predictable deformation and gradual failure, suitable for structures where reliability and serviceability are critical.
5. HPC/UHPC and fiber additives enhance load capacity and crack control, extending the applicability of FRP and hybrids in demanding service conditions.
6. Failure mechanisms of pure FRP are abrupt and brittle, requiring conservative design. Steel and hybrids provide warning signs before failure, improving structural safety.
7. Practical implication: there is no universal solution. The optimal reinforcement depends on project-specific requirements, and design should integrate both mechanical performance and durability considerations.

### 6.2. Potential Directions for Further Research

In the context of the development of reinforcing materials, a number of research areas have been identified, the in-depth analysis of which may contribute to improving the efficiency, durability, and sustainable use of these materials in construction:

1. Materials and structural research, focusing on the microstructure, chemical composition, and effect of heat treatment and surface modifications on the mechanical properties of rebar, is paramount. In the case of composite and hybrid reinforcement, new types of fibers and resins with increased resistance to degradation are necessary, as well as improving the adhesion between the fibers and the matrix, which directly impacts the strength and durability of the reinforcement.
2. Another key area is research on the effects of environmental factors, material fatigue, and extreme impacts such as high humidity, aggressive chemical environments, variable temperatures, and dynamic loads. This requires the development of advanced research methods and numerical models to predict material degradation and their long-term durability.
3. A key research direction is the detailed analysis of damage mechanisms, including crack propagation and interaction between the core (steel or composite) and the outer casing in hybrid reinforcement. New cementitious materials with increased strength and effective transverse reinforcement systems made of composites are also being developed.
4. Work is also underway on creating comprehensive predictive and simulation models that allow the assessment of reinforcement behavior under various loading and environmental conditions. In parallel, life-cycle analysis (LCA/LCC) methods are being developed to assess the cost-effectiveness of implementing specific reinforcement materials, taking into account their environmental impact, CO_2_ emissions, and the consumption of natural resources.
5. It is also essential to focus on the development and harmonization of predictive models for bond and durability of reinforcing materials. For composite and hybrid bars, future research should integrate fatigue and environmental findings and account for long-term interactions with matrices such as UHPC, especially under combined chloride exposure and sustained dynamic loading. Expanding design code provisions to cover hybrid reinforcement and conducting dedicated fire resistance testing are further priorities. These efforts will enable more accurate prediction of reinforcement behavior under extreme conditions and improve the long-term safety and durability of structures.
6. The final area concerns the harmonization of design, implementation, and inspection standards for composite and hybrid reinforcement.

## Figures and Tables

**Table 1:** Summary of strength parameters of steel bars.

Ref.	Diameter [mm]	Elastic Modulus [GPa]	Yield Strength [MPa]	Ultimate Strength [MPa]
[89]	10	200	420	570
	12	200	425	582
	16	200	430	585
[102]	10	200	423	588
[101]	8	200	240	350
	12	200	475	615
[49]	12	200	507	610
[103]	12	200	525	690
	18	200	520	700
	22	200	545	705
[50]	8	198	456	683
	10	198	599	520
[104]	14	215	426	597
[105]	10	200	406	-
[106]	10	199	498	676
[51]	10	195	410	540

**Table 2:** Summary of strength parameters of composite bars.

Ref.	Type of Fiber Used as Reinforcement	Diameter [mm]	Elastic Modulus [GPa]	Ultimate Strength [MPa]
[102]	Carbon	10	192	2610
		10	158	2038
[105]	Carbon	6	169	2701
		10	152	2437
		13	125	2001
[111]	Carbon	12	42	902
		14	43	808
		18	41	963
[101]	Glass	12	40	820
[49]	Glass	12	40	868
		16	46	958
[103]	Glass	12	54	619
		18	49	520
		22	51	570
[50]	Glass	10	64	998
[104]	Glass	14	40	885
[105]	Glass	10	40	1000
[106]	Glass	20	42	846
		10	45	868

**Table 3:** Summary of strength parameters of hybrid bars.

Ref.	Construction Of Hybrid Bar	Core Diameter [mm]	Diameter [mm]	Elastic Modulus [GPa]	Yield Strength [MPa]	Ultimate Strength [MPa]
[90]	SBFCB	8	10.8	175	350	764
		8	11.5	155	309	811
		8	12.1	140	279	939
[51]	SBFCB	6	10	105	222	916
		10	14	126	266	797
[89]	SCFCB	10	10.5	198	432	642
		12	12.5	198	438	610
		12	12.8	198	435	687
[51]	SCFCB	6	10	173	363	1620
[101]	SGFCB	10	14	140	396	700
[51]	SGFCB	6	10	99	208	724
[112]	GFRP-UHPC-GFRP	12	45	44	-	766
		20	45	43	-	700
[113]	GFRP-UHPC-GFRP	12	45	44	-	766
		20	45	44	-	714
		25	45	46	-	668
[113]	GFRP-UHPC-CFRP	20	45	-	-	714
[112]	CFRP-UHPC-CFRP	20	45	142	-	2296

**Table 4:** Summary of mechanical parameters of structural bars.

Type of Bars	Elastic Modulus [GPa]	Yield Strength [MPa]	Ultimate Strength [MPa]
Steel	200	240–600	350–705
CFRP	125–192	-	2000–2700
GFRP	40–64	-	520–1000
SBFCB	105–175	222–350	764–939
SCFCB	173–198	363–438	610–1620
SGFCB	99–140	208–396	700–724
GFRP-UHPC-GFRP	43–46	-	668–766
GFRP-UHPC-CFRP	-	-	714
CFRP-UHPC-GFRP	142	-	2296

**Table 5:** Summary of durability properties of composite bars.

Property/Feature	CFRP	BFRP	GFRP
Temperature resistance	Good; retains 50–80% strength up to 350 °C; strength drops rapidly above 350 °C	Medium; shear strength increases up to 300 °C, then decreases	Weakest; intense degradation of resin and fiber already above 200 °C, carbonization at 300–400 °C
Resistance to alkaline environments	Very good; strength retention approx. 75%, acid resistant	Good; retention 85–95%, favorable cost-performance ratio	Weakest; susceptible to fiber corrosion due to glass reaction
Creep crack resistance	Highest resistance	Good, outperforms GFRP at low stresses	Lowest resistance
Resistance to freeze–thaw cycles	Stable modulus of elasticity; decrease in tensile strength	Elastic modulus stable or increasing; tensile strength increases	Decrease in tensile strength; degradation of elongation at break
Resistance to chloride solutions	Highest; >70% strength retention, up to 160% better than BFRP in aggressive conditions	Good; >70% strength retention	Weakest; only 30–70% strength retention
Cost and availability	Higher cost, best durability	Optimal cost-performance ratio	Lowest cost but limited durability in aggressive conditions

**Table 6:** Summary of beam failure mechanisms in the four-point bending test.

Characteristic	Steel Reinforcement (Figure 7a)	Composite Reinforcement (Figure 7b)	Hybrid Reinforcement (Figure 7c)
The nature of the failure mechanism	Continuous, predictable, malleable	Fragile, sudden, sudden	Intermediate–partially ductile
Damage begins	Small vertical cracks	Deep diagonal cracks	Yield of the steel core, sudden cover fracture
The role of reinforcement in damage control	Controls crack spread, warning signals	Sudden rupture without warning	Some ductility, but no warnings after casing failure
Behavior after cracking	Retains its load-bearing capacity, visible deformations	Sudden breakage	Core yields, outer layer fractures suddenly
Type of concrete damage	Compression zone after yield	Crushing, spalling, shear cracks	Crushing after rod continuity breaks
Influence of transverse reinforcement	Controls crack development (e.g., steel stirrups)	Rapid crack development, stirrups improve control	Depends on the combination of materials
Possibility of predicting failure	High–gradual damage and clear warning signals	Low–sudden, unpredictable	Low–no clear warning signals, sudden failure

**Table 7:** Results of experiments on beams with different types of reinforcement.

Ref.	Compressive Strength [MPa]	Matrix	Fibers [%]	Type of Bars	Reinforcement	F_cr_ [kN]	F_u_ [kN]
[90]	39	NC	-	Steel bars	3#8	63	111
				Steel bars	2#8	26	35
				Steel bars	2#8	23	38
				Steel bars + SBFCB	2#8 + 1#11.5-8	84	145
				Steel bars + SBFCB	1#8 + 2#11.5-8	99	168
				SBFCB	3#11.5-8	106	203
				SBFCB	2#10.8-8	48	65
				SBFCB	2#11.5-8	42	64
				SBFCB	2#12.1-8	49	77
				SBFCB	2#12.1-8	47	74
[89]	30	NC	-	Steel bars	3#10	36	89
				Steel bars	3#12	37	135
				Steel bars	3#16	38	253
	40			Steel Bars	3#12	43	147
				Steel bars	3#16	45	279
	50			Steel bars	3#12	48	159
				Steel bars	3#16	51	290
	30			SCFCB	2#10.5	36	93
				SCFCB	2#12.5	38	141
				SCFCB	2#12.8	38	260
	40			SCFCB	2#12.5	44	152
				SCFCB	2#12.8	46	275
	50			SCFCB	2#12.5	49	160
				SCFCB	2#12.8	52	288
[101]	30	NC	-	Steel bars	3#12	22	110
			1 PP+ 1 MPP	Steel bars	3#12	29	140
				Steel bars	6#12	33	166
				Steel bars + GFRP	3#12 + 3#12	31	200
			-	GFRP	3#12	18	96
			1 PP+ 1 MPP	GFRP	3#12	28	113
				SGFCB + GFRP	2#14 + 3#12	42	195
				SGFCB	2#14	29	131
			-	SGFCB	3#14	20	147
			0.5 PP+ 0.5 MPP	SGFCB	3#14	30	168
			1 PP+ 1 MPP	SGFCB	3#14	32	175
				SGFCB	4#14	31	185
				SGFCB	3#14	28	80
				SGFCB	3#14	32	222
[49]	50	NC	0.5 SF	Steel bars	4#12	56	274
				Steel bars	4#12	52	262
				GFRP	4#12	45	300
				GFRP + steel bars	2#12 + 2#12	52	302
				GFRP + steel bars	2#16 + 2#12	51	325
				GFRP + steel bars	2#12 + 2#12	50	271
				GFRP + steel bars	2#16 + 2#12	40	278
[104]	30	NC	-	Steel bars + steel stirrups	2#14 + #6 co 9 cm	26	52
				GFRP	2#14	21	31
				GFRP + carbon stirrups	2#14 + #20 co 9 mm	24	36
				GFRP + carbon stirrups	2#14 + #20 co 9 mm	25	51
				GFRP + carbon stirrups	2#14 + #20 co 6 mm	29	56
				GFRP + carbon stirrups	2#14 + #20 co 9 mm	26	53
				GFRP + carbon stirrups	2#14 + #20 co 12 mm	24	48
[112]	25	NC	-	CFRP + (GFRP-UHPC-GFRP)	2#20 + 2#45	9	166
				CFRP + (GFRP-UHPC-GFRP)	3#20 + 2#45	9	188
	40			CFRP + GFRP	2#20 + 2#12	6	113
				CFRP + GFRP	3#20 + 2#12	9	123
				CFRP + (GFRP-UHPC-GFRP)	2#20 + 2#45	9	160
				CFRP + (GFRP-UHPC-GFRP)	2#20 + 2#45	8	208
				CFRP + (GFRP-UHPC-GFRP)	3#20 + 2#45	8	172
				CFRP + (GFRP-UHPC-GFRP)	3#20 + 2#45	6	190
				CFRP-UHPC-GFRP + GFRP-UHPC-GFRP	2#45 + 2#45	9	103
[113]	55	NC	-	GFRP + GFRP	3#25 + 2#12	18	248
				GFRP + GFRP-UHPC-GFRP	3#25 + 2#45	21	328
				GFRP + GFRP-UHPC-GFRP	3#25 + 2#45	21	357
				GFRP + GFRP-UHPC-GFRP	3#25 + 2#45	24	359
				GFRP + GFRP-UHPC-GFRP	2#25 + 2#45	21	243
				GFRP + GFRP-UHPC-GFRP	4#25 + 2#45	18	338
				GFRP + GFRP-UHPC-CFRP	3#25 + 2#45	18	348
[102]	40	NC	-	CFRP (rib depth 0.2 mm)	1#10	55	131
				CFRP (rib depth 0.2 mm)	3#10	121	333
				CFRP (rib depth 0.6 mm)	1#10	45	117
				CFRP (rib depth 0.6 mm)	3#10	114	331
	40 + UHDC	NC + UHDC		CFRP (rib depth 0.2 mm)	1#10	63	150
				CFRP (rib depth 0.2 mm)	3#10	121	382
				CFRP (rib depth 0.6 mm)	1#10	54	135
				CFRP (rib depth 0.6 mm)	3#10	112	370
[50]	30	NC	-	Steel bars	3#10	15	65
				Steel bars + GFRP	1#10 + 2#10	12	79
				Steel bars + GFRP	2#10 + 1#10	14	65
				GFRP	3#10	10	84
	140	UHPC	1 SF	Steel bars	3#10	6	71
				Steel bars + GFRP	1#10 + 2#10	11	95
				Steel bars + GFRP	2#10 + 1#10	16	80
				GFRP	3#10	12	99
[103]	120	UHPC	2 SF	Steel bars	2#12	59	226
				Steel bars	2#18	61	399
				Steel bars	2#22	65	529
				GFRP	2#12	56	185
				GFRP	2#18	59	303
				GFRP	2#22	62	493
[111]	159	UHPC	2 SF	CFRP	4#12	7	85
				CFRP	3#14	6	78
				CFRP	2#18	6	73
				CFRP	3#14	7	83
				CFRP	3#12	7	68
				CFRP	3#12	5	57
[105]	46	NC	-	CFRP	3#10	14	126
				GFRP	3#10	13	90
	45 ECC	ECC	2 PVA	Steel bars	3#10	14	83
				CFRP	2#10	11	121
				CFRP	3#10	13	152
				CFRP	5#10	13	179
				CFRP	3#6	11	96
				CFRP	3#13	14	182
				GFRP	3#10	12	103
[106]	51	NC	-	GFRP + glass stirrups	2#20 + #10 co 10 cm	33	262
	51	NC	-	GFRP + steel bars + glass stirrups	2#20 + 1#20 + #10 co 8 cm	50	376
	51 + 46 ECC	NC + ECC	2 PE	GFRP + glass stirrups	2#20 + #10 co 10 cm	20	281
	51 + 46 ECC	NC + ECC		GFRP + glass stirrups	3#20 + #10 co 8 cm	30	354
	51 + 46 ECC	NC + ECC		GFRP + glass stirrups	4#20 + #10 co 6 cm	40	341
	51 + 46 ECC	NC + ECC	2 PE	GFRP + steel bars + glass stirrups	2#20 + 1#20 + #10 co 8 cm	38	383
	46 ECC	ECC	2 PE	GFRP + glass stirrups	2#20 + #10 co 10 cm	18	346

Note. To maintain comparability, Table 7 lists baseline configurations only. Dosage-dependent effects of polypropylene micro/macro-fibers are summarized in the text for steel, GFRP, and SGFCB (Abdel-Karim et al. [101]).

**Table 8:** Comparison of the load-bearing capacity of reinforced beams with different types of composite bars.

Ref.	Compressive Strength [MPa]	Matrix	Fibers [%]	Type of Fiber Used as Reinforcement	Reinforcement	F_cr_ [kN]	F_u_ [kN]
[102]	40	NC	-	CFRP	1#10	55	131
[102]	40	NC	-		1#10	45	117
[102]	40	NC	-		3#10	121	333
[102]	40	NC	-		3#10	114	331
[105]	46	NC	-		3#10	14	126
[105]	45 ECC	ECC	2 PVA		2#10	11	121
[105]	45 ECC	ECC	2 PVA		3#10	13	152
[105]	45 ECC	ECC	2 PVA		5#10	13	179
[105]	45 ECC	ECC	2 PVA		3#6	11	96
[105]	45 ECC	ECC	2 PVA		3#13	14	182
[102]	40 + UHDC	NC + UHDC	-		1#10	63	150
[102]	40 + UHDC	NC + UHDC	-		1#10	54	135
[102]	40 + UHDC	NC + UHDC	-		3#10	121	382
[102]	40 + UHDC	NC + UHDC	-		3#10	112	370
[111]	159	UHPC	SF		4#12	7	85
[111]	159	UHPC	SF		3#14	6	78
[111]	159	UHPC	SF		2#18	6	73
[111]	159	UHPC	SF		3#14	7	83
[111]	159	UHPC	SF		3#12	7	68
[111]	159	UHPC	SF		3#12	5	57
[50]	30	NC	-	GFRP	3#10	10	84
[101]	30	NC	-		3#12	18	96
[104]	30	NC	-		2#14	21	31
[105]	46	NC	-		3#10	13	90
[101]	30	NC	1 PP + 1 MPP		3#12	28	113
[49]	50	NC	0.5 SF		4#12	45	300
[105]	45 ECC	ECC	2 PVA		3#10	12	103
[103]	120	UHPC	2 SF		2#12	56	185
[103]	120	UHPC	2 SF		2#18	59	303
[103]	120	UHPC	2 SF		2#22	62	493
[50]	140	UHPC	1 SF		3#10	12	99
[113]	55	NC	-	GFRP + GFRP	3#25 + 2#12	18	248
[104]	30	NC	-	GFRP + carbon stirrups	2#14 + #20 co 9 mm	24	36
[104]	30	NC	-		2#14 + #20 co 9 mm	25	51
[104]	30	NC	-		2#14 + #20 co 6 mm	29	56
[104]	30	NC	-		2#14 + #20 co 9 mm	26	53
[104]	30	NC	-		2#14 + #20 co 12 mm	24	48
[106]	51	NC	-	GFRP + glass stirrups	2#20 + #10 co 10 cm	33	262
[106]	51 + 46 ECC	ECC	2 PE		2#20 + #10 co 10 cm	20	281
[106]	46 ECC	ECC	2 PE		2#20 + #10 co 10 cm	18	346
[106]	51 + 46 ECC	ECC	2 PE		3#20 + #10 co 8 cm	30	354
[106]	51 + 46 ECC	ECC	2 PE		4#20 + #10 co 6 cm	40	341

**Table 9:** Comparison of the load-bearing capacity of reinforced beams with different types of hybrid bars.

Ref.	Compressive Strength [MPa]	Matrix	Fibers [%]	Type of Bars	Reinforcement	F_cr_ [kN]	F_u_ [kN]
[90]	30	NC	-	SBFCB	3#11.5-8	106	203
					2#10.8-8	48	65
					2#11.5-8	42	64
					2#12.1-8	49	77
					2#12.1-8	47	74
[89]	30			SCFCB	2#10.5	36	93
					2#12.5	38	141
					2#12.8	38	260
	40				2#12.5	44	152
					2#12.8	46	275
	50				2#12.5	49	160
					2#12.8	52	288
[101]	30			SGFCB	3#14	20	147
			0.5 PP + 0.5 MPP		3#14	30	168
			1 PP + 1 MPP		2#14	29	131
			1 PP + 1 MPP		3#14	32	175
			1 PP + 1 MPP		3#14	28	80
			1 PP + 1 MPP		4#14	31	185
[112]	40		-	CFRP-UHPC-GFRP + GFRP-UHPC-GFRP	2#45 + 2#45	9	103

**Table 10:** Summary of the load-bearing capacity of beams reinforced with various types of bars.

Ref.	Compressive Strength [MPa]	Matrix	Fibers [%]	Type of Bars	Reinforcement	F_cr_ [kN]	F_u_ [kN]
[89]	30	NC	-	SCFCB	2#10.5	36	93
	30			SCFCB	2#12.5	38	141
	30			SCFCB	2#12.8	38	260
	40			SCFCB	2#12.5	44	152
	40			SCFCB	2#12.8	46	275
	50			SCFCB	2#12.5	49	160
	50			SCFCB	2#12.8	52	288
[102]	40	NC	-	SCFCB (rib depth 0.2 mm)	1#10	55	131
	40			SCFCB (rib depth 0.6 mm)	1#10	45	117
	40			SCFCB (rib depth 0.2 mm)	3#10	121	333
	40			SCFCB (rib depth 0.6 mm)	3#10	114	331
	40 + UHDC	NC + UHDC		SCFCB (rib depth 0.2 mm)	1#10	63	150
	40 + UHDC			SCFCB (rib depth 0.6 mm)	1#10	54	135
	40 + UHDC			SCFCB (rib depth 0.2 mm)	3#10	121	382
	40 + UHDC			SCFCB (rib depth 0.6 mm)	3#10	112	370
[105]	40	NC	-	CFPR	3#10	14	126
	45 ECC	ECC	2 PVA	CFPR	2#10	11	121
	45 ECC			CFPR	3#10	13	152
	45 ECC			CFPR	5#10	13	179
	45 ECC			CFPR	3#6	11	96
	45 ECC			CFPR	3#13	14	182
[111]	159	UHPC	SF	CFPR	4#12	7	85
	159			CFPR	3#14	6	78
	159			CFPR	2#18	6	73
	159			CFPR	3#14	7	83
	159			CFPR	3#12	7	68
	159			CFPR	3#12	5	57
[112]	40	NC	-	CFRP + GFRP	2#20 + 2#12	6	113
	40			CFRP + GFRP	3#20 + 2#12	9	123
	25			CFRP + (GFRP-UHPC-GFRP)	2#20 + 2#45	9	166
	25			CFRP + (GFRP-UHPC-GFRP)	3#20 + 2#45	9	188
	40			CFRP + (GFRP-UHPC-GFRP)	2#20 + 2#45	9	160
	40			CFRP + (GFRP-UHPC-GFRP)	2#20 + 2#45	8	208
	40			CFRP + (GFRP-UHPC-GFRP)	3#20 + 2#45	8	172
	40			CFRP + (GFRP-UHPC-GFRP)	3#20 + 2#45	6	190
	40			CFRP-UHPC-GFRP + GFRP-UHPC-GFRP	2#45 + 2#45	9	103

**Table 11:** Summary of the load-bearing capacity of beams reinforced with various types of bars.

Ref.	Compressive Strength [MPa]	Matrix	Fibers [%]	Type of Bars	Reinforcement	F_cr_ [kN]	F_u_ [kN]
[101]	30	NC	-	SGFCB	3#14	20	147
	30		0.5 PP + 0.5 MPP	SGFCB	3#14	30	168
	30		1 PP + 1 MPP	SGFCB	2#14	29	131
	30			SGFCB	3#14	32	175
	30			SGFCB	3#14	28	80
	30			SGFCB	3#14	32	222
	30			SGFCB	4#14	31	185
	30			SGFCB + GFRP	2#14 + 3#12	42	195
[101]	30	NC	-	GFRP	3#12	18	96
[104]	30			GFRP	2#14	21	31
[105]	46			GFRP	3#10	13	90
[50]	30		3 SF	GFRP	3#10	10	84
[101]	30		1 PP + 1 MPP	GFRP	3#12	28	113
[49]	50		0.5 SF	GFRP	4#12	45	300
[105]	45 ECC	ECC	2 PVA	GFRP	3#10	12	103
[103]	120	UHPC	2 SF	GFRP	2#12	56	185
[103]	120			GFRP	2#18	59	303
[103]	120			GFRP	2#22	62	493
[50]	140		6 SF	GFRP	3#10	12	99
[104]	30	NC	-	GFRP + carbon stirrups	2#14 + #20 co 9 mm	24	36
	30			GFRP + carbon stirrups	2#14 + #20 co 9 mm	25	51
	30			GFRP + carbon stirrups	2#14 + #20 co 6 mm	29	56
	30			GFRP + carbon stirrups	2#14 + #20 co 9 mm	26	53
	30			GFRP + carbon stirrups	2#14 + #20 co 12 mm	24	48
[106]	51	NC	-	GFRP + glass stirrups	2#20 + #10 co 10 cm	33	262
	51 + 46 ECC	NC + ECC	2 PE	GFRP + glass stirrups	2#20 + #10 co 10 cm	20	281
	46 ECC	ECC		GFRP + glass stirrups	2#20 + #10 co 10 cm	18	346
	51 + 46 ECC	NC + ECC		GFRP + glass stirrups	3#20 + #10 co 8 cm	30	354
	51 + 46 ECC	NC + ECC		GFRP + glass stirrups	4#20 + #10 co 6 cm	40	341
[113]	55	NC	-	GFRP + GFRP	3#25 + 2#12	18	248
	55			GFRP + GFRP-UHPC-GFRP	3#25 + 2#45	21	328
	55			GFRP + GFRP-UHPC-GFRP	3#25 + 2#45	21	357
	55			GFRP + GFRP-UHPC-GFRP	3#25 + 2#45	24	359
	55			GFRP + GFRP-UHPC-GFRP	2#25 + 2#45	21	243
	55			GFRP + GFRP-UHPC-GFRP	4#25 + 2#45	18	338
	55			GFRP + GFRP-UHPC-CFRP	3#25 + 2#45	18	348

## Data Availability

No new data were created or analyzed in this study. Data sharing is not applicable to this article.
